# Performance of AI-Based Screening Tools for Obstructive Sleep Apnea Across Apnea-Hypopnea Index Thresholds: Systematic Review and Meta-Analysis

**DOI:** 10.2196/92399

**Published:** 2026-09-11

**Authors:** Yujia Lv, Lihui Zhou, Sihan Jiao, Jiaying Lin, Boran Sun, Haixia Hao, Chenxiao Jia, Yuan Wang, Li Bu, Wenli Lu

**Affiliations:** 1 Department of Epidemiology and Health Statistics, School of Public Health, Tianjin Medical University, 22 Qixiangtai Road, Heping District, Tianjin, 300070, China, 86 022-83336619; 2 Tianjin Key Laboratory of Environment, Nutrition and Public Health, Tianjin Medical University, Tianjin, China; 3 Laboratory for Artificial Intelligence and Active Health, Tianjin Medical University, Tianjin, China; 4 Jinqiu Hospital of Liaoning Province, Shenyang, China

**Keywords:** obstructive sleep apnea, artificial intelligence, screening, systematic review, meta-analysis

## Abstract

**Background:**

Obstructive sleep apnea (OSA) is highly prevalent but remains substantially underdiagnosed. Polysomnography (PSG) is the reference standard, but its cost and limited availability constrain large-scale case identification. AI-based screening tools may support risk stratification and referral prioritization, but their diagnostic accuracy across apnea-hypopnea index (AHI) thresholds remains uncertain.

**Objective:**

This review aimed to systematically evaluate the diagnostic accuracy of AI-based OSA screening tools at AHI thresholds of ≥5, ≥15, and ≥30 events/hour, with emphasis on models using non-PSG–derived inputs.

**Methods:**

PubMed, Embase, Scopus, and Web of Science were searched for studies published from January 1, 2016, to May 3, 2026. Eligible studies included adults evaluated for suspected OSA or recruited from population-based cohorts, assessed AI-based models intended or interpretable for OSA screening, risk prediction, or screening-oriented severity classification, used PSG as the reference standard, and reported sufficient data to construct or reconstruct 2×2 contingency tables. Diagnostic accuracy was synthesized separately by AHI threshold and input source using bivariate random-effects models, with 95% CIs and prediction intervals (PIs). Risk of bias and certainty of evidence were assessed using QUADAS-2 (Quality Assessment of Diagnostic Accuracy Studies 2) and GRADE (Grading of Recommendations Assessment, Development, and Evaluation), respectively.

**Results:**

A total of 60 studies were included, of which 47 contributed data to the meta-analysis. At AHI thresholds of ≥5, ≥15, and ≥30 events/hour, pooled sensitivities were 0.94 (95% CI 0.92‐0.96; 95% PI 0.71‐0.99), 0.87 (95% CI 0.84‐0.89; 95% PI 0.66‐0.96), and 0.83 (95% CI 0.79‐0.87; 95% PI 0.61‐0.94), respectively; the corresponding specificities were 0.77 (95% CI 0.69‐0.84; 95% PI 0.30‐0.96), 0.81 (95% CI 0.75‐0.85; 95% PI 0.39‐0.96), and 0.91 (95% CI 0.87‐0.94; 95% PI 0.55‐0.99), respectively. The corresponding areas under the summary receiver operating characteristic curves were 0.943, 0.907, and 0.920. For non-PSG–derived tools, sensitivities were 0.92, 0.85, and 0.81, and specificities were 0.70, 0.74, and 0.85 at the 3 thresholds, respectively. For PSG-derived models, sensitivities were 0.96, 0.90, and 0.85, and specificities were 0.82, 0.88, and 0.96, respectively. Exploratory subgroup analyses suggested performance variation across selected study and model characteristics, including region, algorithmic framework, data source, and validation method.

**Conclusions:**

AI-based tools showed generally favorable screening performance for OSA across clinically relevant AHI thresholds, although wide PIs suggest variable performance across future comparable populations and settings. By synthesizing diagnostic accuracy across 3 AHI thresholds and distinguishing non-PSG–derived from PSG-derived models, this review extends previous broad or modality-specific reviews and offers a clinically interpretable, pathway-specific basis for linking model performance to intended use. The findings may clarify potential roles for non-PSG–derived tools in front-end screening and referral prioritization and for PSG-derived models in reduced-channel assessment and sleep-laboratory workflow support. Given substantial heterogeneity, limited external validation, and low or very low certainty of evidence, prospective validation is needed before routine implementation.

## Introduction

Obstructive sleep apnea (OSA) is a highly prevalent sleep-related breathing disorder characterized by recurrent upper airway obstruction during sleep [1]. These events lead to intermittent hypoxemia, intrathoracic pressure swings, and sleep fragmentation [2,3]. The global burden of OSA is substantial [4]. A large population-based analysis estimated that approximately 936 million adults aged 30‐69 years worldwide are affected by mild-to-severe OSA, including 425 million with moderate-to-severe disease [5]. Projection modeling from the United States suggests that the burden may continue to increase over the coming decades [6]. Despite its high prevalence, an estimated 80% to 90% of affected adults remain undiagnosed and untreated [7,8]. This diagnostic gap carries significant clinical and societal consequences. OSA is associated with cardiovascular disease, metabolic dysfunction, neurocognitive impairment, and impaired daytime alertness, which may increase the risk of motor vehicle and occupational accidents [9-11].

Polysomnography (PSG) is considered the reference standard for OSA diagnosis because it provides a comprehensive assessment of respiratory events and sleep physiology; however, its high cost and limited availability restrict its use for large-scale case identification [12,13]. Home sleep apnea testing (HSAT) improves accessibility but may underestimate the apnea-hypopnea index (AHI), particularly in mild OSA, because it often lacks electroencephalography and relies on recording time rather than objectively measured sleep time [14]. As a result, large-scale identification of individuals at risk of OSA remains difficult [15]. Initial screening often relies on symptoms, clinical risk factors, and questionnaire-based tools or clinical prediction models, which are practical but show variable performance across populations and disease severity [16,17]. More effective and scalable screening strategies are therefore needed to support early identification, risk stratification, and referral for confirmatory diagnosis and treatment [15,18].

Advances in AI, particularly machine learning and deep learning, have provided new approaches for OSA screening and early risk stratification [19,20]. AI-based models can identify OSA-related patterns from clinical variables, physiological signals, imaging data, acoustic recordings, wearable sensors, or multimodal data, thereby offering a scalable approach to risk assessment and referral prioritization. However, these tools should be viewed as adjuncts to, rather than replacements for, confirmatory diagnostic testing with PSG or HSAT [21,22].

Although recent reviews have summarized the expanding literature on AI applications in OSA [20-23], evidence remains limited regarding the threshold-specific screening performance of AI-based models. Existing studies differ substantially in populations, data sources, model architectures, validation strategies, reference standards, and outcome definitions. Moreover, many reviews have been narrative, scoping, or modality-specific [20-22], with limited quantitative synthesis of diagnostic accuracy [23]. Threshold-specific evidence is particularly needed because OSA severity is commonly defined by AHI thresholds of ≥5, ≥15, and ≥30 events/hour, across which model performance may vary. The clinical applicability of AI models may also differ according to whether inputs are derived from PSG signals or from more accessible non-PSG sources. These methodological and reporting limitations—including limited external validation, unclear risk of bias, and incomplete reporting of diagnostic count data—support the need for further quantitative synthesis of AI-based OSA screening performance across clinically relevant AHI thresholds and input sources.

Therefore, this review aimed to evaluate the diagnostic accuracy of AI-based screening tools for identifying individuals at risk of any OSA (AHI ≥5 events/h), moderate-to-severe OSA (AHI ≥15 events/h), and severe OSA (AHI ≥30 events/h), with primary emphasis on models using non-PSG–derived inputs. Models using PSG-derived signals were analyzed separately because they represent a different clinical implementation pathway. We also performed exploratory subgroup analyses to investigate potential sources of interstudy heterogeneity.

## Methods

### Overview

This systematic review and meta-analysis was reported in accordance with the PRISMA 2020 (Preferred Reporting Items for Systematic Reviews and Meta-Analyses 2020) statement [24] and its extension for Diagnostic Test Accuracy Studies (PRISMA-DTA) [25]. The literature search was reported in accordance with the PRISMA literature search extension (PRISMA-S) [26]. The completed PRISMA 2020 expanded, PRISMA-DTA, and PRISMA-S checklists are provided in Checklist 1. This review was registered with the International Prospective Register of Systematic Reviews (PROSPERO; registration number CRD420251271773).

### Search Strategy

Two researchers independently conducted the literature searches and record screening. Disagreements were resolved by a third reviewer with expertise in data analysis. All searches were performed in PubMed, Embase, Scopus, and Web of Science on May 3, 2026, and were limited to studies published within the preceding 10 years. The search strategy combined database-specific controlled vocabulary terms, where available, and free-text keywords related to 3 core concepts: obstructive sleep apnea, AI-based methods, and screening or diagnostic classification. Search terms included synonyms and variants for obstructive sleep apnea, sleep-disordered breathing, AI, machine learning, deep learning, neural networks, computer vision, and diagnostic performance. To ensure comprehensiveness, backward and forward citation searching of included studies was conducted on June 23, 2026, to identify additional eligible studies. The search was conducted without language restrictions. The search strategy was reviewed by the study team before implementation. The full search strategies for each database are provided in Table S1 in Multimedia Appendix 1.

### Study Eligibility Criteria

We included original studies involving adults aged 18 years or older who were evaluated for suspected OSA or recruited from population-based cohorts. Eligible studies evaluated AI-based models, including machine learning or deep learning algorithms, that used clinical variables, physiological signals, acoustic recordings, imaging data, or multimodal inputs for OSA screening or screening-oriented classification. Studies described as diagnostic or severity-classification models were also eligible if their outputs could be interpreted in relation to OSA presence or severity and were applicable to screening-oriented evaluation. Eligible studies used PSG as the reference standard and defined OSA according to standard AHI thresholds. Studies with a total sample size of fewer than 50 participants were excluded. For quantitative synthesis, studies were required to provide sufficient diagnostic accuracy data to extract or reconstruct true-positive (TP), false-positive (FP), true-negative (TN), and false-negative (FN) counts. Studies with incomplete or nonextractable diagnostic accuracy data were excluded from quantitative synthesis but summarized narratively when relevant.

### Study Selection

Duplicate records were removed using EndNote X9 (Clarivate). Two reviewers independently screened titles and abstracts, and full texts of potentially relevant studies were subsequently assessed for eligibility against the predefined inclusion criteria. Any disagreements were resolved by discussion or consultation with a third reviewer.

### Data Extraction

Two independent reviewers extracted data in duplicate using standardized extraction forms, with disagreements resolved by consensus or consultation with a third reviewer. Extracted information included study characteristics, participant characteristics, AI model type, input modality, validation strategy, data source, reference standard, and AHI or respiratory disturbance index (RDI) thresholds used to define OSA.

We classified input sources as PSG-derived when the AI model used physiological signals collected during PSG or from PSG databases and as non-PSG–derived when inputs were obtained from portable, wearable, questionnaire-based, demographic, or other non-PSG sources. Diagnostic accuracy data were extracted for each reported threshold, including sensitivity, specificity, area under the receiver operating characteristic curve (AUC), and 2×2 contingency table data where available. When TP, FP, TN, and FN were not directly reported, they were reconstructed where possible from confusion matrices, reported sensitivity and specificity with corresponding numbers of OSA-positive and OSA-negative participants, or multiclass severity matrices converted into binary classifications at AHI ≥5, ≥15, and ≥30 events/hour. Reconstructed values were checked against the reported sample size, prevalence, sensitivity, and specificity for consistency. Studies without directly extractable or reliably reconstructable 2×2 data were excluded from quantitative synthesis but summarized narratively when relevant.

Some studies defined OSA using RDI rather than AHI. These data were included only when equivalent event-per-hour thresholds were reported, and the RDI-based definition was considered clinically comparable to the corresponding AHI-based threshold. RDI-based definitions were noted during extraction as a potential source of heterogeneity. When multiple thresholds were reported, data were extracted separately for each threshold and included only in the corresponding threshold-specific analysis. When multiple cohorts, splits, or validation datasets were reported within 1 study, we selected one representative dataset-model combination for analysis. Priority was given to the author-defined primary, final, or recommended model evaluated in an external or independent validation cohort. If no such model or cohort was specified, we selected the model with complete diagnostic data from the largest or most clinically representative test set with the lowest risk of data leakage.

### Quality Assessment and Certainty of Evidence

Risk of bias and applicability concerns were assessed using the QUADAS-2 (Quality Assessment of Diagnostic Accuracy Studies 2) tool [27]. Two reviewers independently performed the assessment, with disagreements resolved by consensus. Studies were classified as high quality if no major domain was rated as high risk of bias, and no substantial applicability concerns were identified; otherwise, they were classified as low quality. PSG-derived AHI or RDI was considered an appropriate reference standard when clearly defined and reported. RDI-based definitions were not automatically judged as low or high risk; judgments depended on whether the reference standard was clearly defined, clinically appropriate, and applicable to the AHI-based target condition of this review.

We used the GRADE (Grading of Recommendations, Assessment, Development, and Evaluation) framework for diagnostic test accuracy studies to evaluate the certainty of evidence for the pooled sensitivity and specificity of AI-based screening tools for OSA across AHI thresholds and input-source categories. The assessment considered 5 domains: risk of bias, indirectness, inconsistency, imprecision, and publication bias. Certainty ratings were classified as high, moderate, low, or very low. AUC was calculated and reported as an additional measure of overall diagnostic performance. The GRADE summary of findings table was prepared using the diagnostic test accuracy framework recommended by the GRADE working group [28].

### Data Synthesis and Statistical Analysis

An overall analysis was conducted across all screening-oriented AI models, irrespective of input source. Models were then analyzed separately according to whether their inputs were PSG-derived or non-PSG–derived. The primary analysis focused on non-PSG–derived models because these models are more directly applicable to front-end screening before formal sleep testing. PSG-derived models were evaluated in secondary analyses because they represent a different clinical implementation pathway.

Extracted or reconstructed diagnostic count data were used to estimate sensitivity and specificity. Analyses were conducted separately for prespecified AHI thresholds of ≥5, ≥15, and ≥30 events/hour. When a study reported multiple thresholds, each threshold-specific dataset was included only in the corresponding analysis to avoid double counting within any single meta-analysis. A continuity correction of 0.5 was applied to zero cells in 2×2 tables to enable model estimation.

Pooled estimates of sensitivity, specificity, and diagnostic odds ratios (DORs) with corresponding 95% CIs were calculated. To further characterize between-study heterogeneity and the expected variability of diagnostic performance across different populations and clinical settings, 95% prediction intervals (PIs) for sensitivity and specificity were calculated when at least 3 studies were available for a given analysis [29]. Whereas 95% CIs describe the uncertainty around the pooled average estimates, 95% PIs incorporate between-study heterogeneity and estimate the range within which the sensitivity or specificity of a comparable future study would be expected to fall. Forest plots of study-specific and pooled sensitivity and specificity, as well as summary receiver operating characteristic (SROC) curves, were generated for each AHI threshold. Summary AUCs were estimated from model-based SROC curves.

Threshold effects due to varying model decision cutoffs were not formally assessed because model cutoffs were inconsistently reported and were not extracted as a standardized variable.

Subgroup analyses were performed to explore potential sources of heterogeneity across studies. Given the small number of studies in several subgroups, these analyses were interpreted as exploratory. Robustness was assessed using leave-one-out sensitivity analyses, and small-study effects were evaluated using the Deeks funnel plot asymmetry test. All analyses were performed using R software version 4.3.2 (R Foundation for Statistical Computing) with relevant statistical packages [30].

## Results

### Search Results

**Figure 1.**
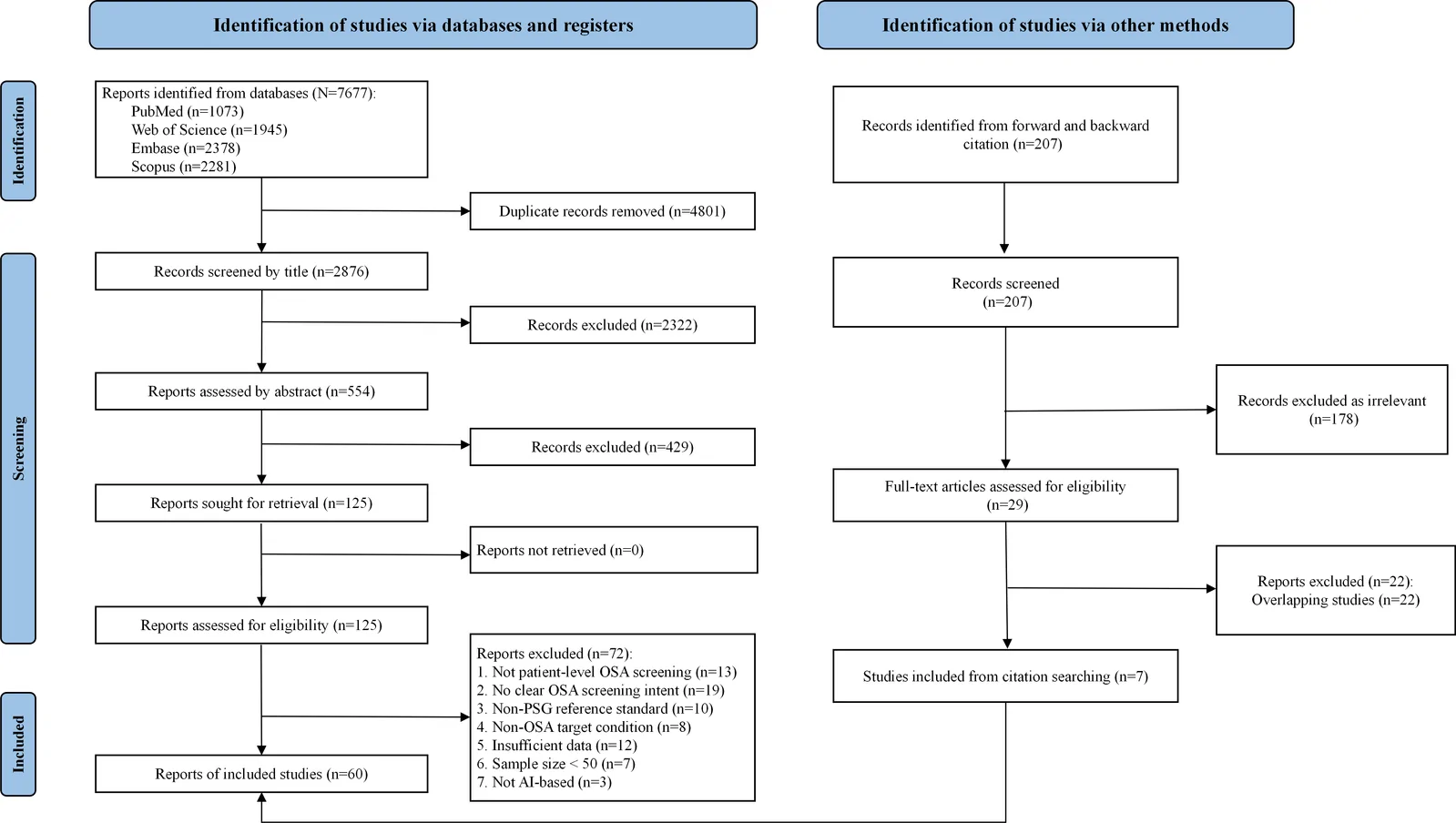
PRISMA (Preferred Reporting Items for Systematic Reviews and Meta-Analyses) flowchart of the study selection process. OSA: obstructive sleep apnea; PSG: polysomnography.

Figure 1 shows the study selection process and results. A total of 7677 records were identified through database searches. After removal of duplicate records (n=4801), 2876 records were screened based on titles, of which 2322 were excluded due to irrelevance. Subsequently, 554 reports were assessed by abstract, and 429 were excluded. A total of 125 full-text articles were assessed for eligibility, and 72 reports were excluded for the following reasons: not patient-level OSA screening (n=13), no clear OSA screening intent (n=19), use of a non-PSG reference standard (n=10), non-OSA target condition (n=8), insufficient data (n=12), sample size <50 (n=7), and not AI-based (n=3). Thus, 53 studies were identified through database searches. Backward and forward citation searching identified 7 additional eligible studies. In total, 60 studies [31-90] were included in the systematic review, and 47 of them were eligible for meta-analyses [31-35,37-40,42,46,48-51,53,55-67,69,72-74,76-78,80-90].

### Characteristics of Included Studies

Table 1 summarizes the characteristics of the 60 included studies [31-90]. The mean sample size was 2626.2 (SD 4488.8; range 60‐24,660) participants. The mean age of participants, available in 48 studies [31-34,36-38,40-54,56-61,64-68,70,72,76-79,81,83-90], was 48.2 (SD 7.0; range 37.3‐70.5) years, and the mean proportion of male participants, reported in 47 studies [31-33,36-61,64-68,70,76-78,81,83-90], was 70% (SD 13.9%; range 40.5%‐100.0%). The studies were most frequently conducted in China (18/60, 30%) [36,38-40,43,44,48,62,64,65,69,78,80,85-88,90], followed by the United States (10/60, 17%) [47,51,58,63,70-73,75,77], Taiwan (9/60, 15%) [35,50,52,53,59,60,66,76,79], and South Korea (8/60, 13%) [37,45,46,54,56,57,83,84]. Most studies used hospital-based data sources (42/60, 70%) [31,34,36,38-48,50,52-58,62,64,67-69,74-79,81-83,85-90] and proprietary datasets (44/60, 73%) [31-34,36,37,40-50,52-62,64,67-69,75-79,81,83-88]. In terms of model type, convolutional neural networks (12/60, 20%) [35,47,48,56,62,69,72,78,83-86] and feed-forward neural networks (12/60, 20%) [38,44,46,49,52,54,55,58,59,61,64,73] were the most common AI approaches, followed by tree-based ensemble models (9/60, 15%) [34,36,60,65,68,75,79,81,90]. A total of 23 out of 60 (38%) studies [31,32,34,35,38,41,54,60-64,69,71-73,80,84-89] used model inputs derived from PSG-recorded channels, whereas 37 out of 60 (62%) studies [33,36,37,39,40,42-53,55-59,65-68,70,74-79,81-83,90] used inputs obtained independently of PSG recordings.

**Table 1.** Characteristics of the included studies (N=60).

Features	Studies	References
Year of publication, n (%)		
2026	5 (8.3)	[56,65,82,83,89]
2025	9 (15.0)	[41,43,61-63,71,74,88,90]
2024	11 (18.3)	[34,38,39,44,46,58,60,69,72,84,87]
2023	9 (15.0)	[35,36,45,53,54,67,75,81,86]
2022	9 (15.0)	[37,48,49,51,59,68,76,78,80]
2021	6 (10.0)	[40,47,52,64,70,85]
2020	3 (5.0)	[31,50,55]
2019	2 (3.3)	[32,42]
2017	4 (6.7)	[33,57,66,73]
2016	2 (3.3)	[77,79]
Country of study, n (%)		
China	18 (30.0)	[36,38-40,43,44,48,62,64,65,69,78,80,85-88,90]
United States	10 (16.7)	[47,51,58,63,70-73,75,77]
Taiwan	9 (15.0)	[35,50,52,53,59,60,66,76,79]
South Korea	8 (13.3)	[37,45,46,54,56,57,83,84]
Brazil	2 (3.3)	[32,41]
Others (<2)	12 (20.0)	[31,33,34,42,49,55,61,67,68,74,81,82]
Not reported	1 (1.7)	[89]
Sample size		
Total participants, n	157,574	[31-90]
Value, mean (SD; range)	2626.2 (4488.8; 60.0‐24660.0)	[31-90]
Male participants (%)		
Value, mean (SD; range)	70.0 (13.9; 40.5‐100.0)	[31-33,36-61,64-68,70,76-78,81,83-90]
Not reported, n (%)	13 (21.7)	[34,35,62,63,69,71-75,79,80,82]
Age (y)		
Value, mean (SD; range)	48.2 (7.0; 37.3‐70.5)	[31-34,36-38,40-54,56-61,64-68,70,72,76-79,81,83-90]
Not reported, n (%)	12 (20.0)	[35,39,55,62,63,69,71,73-75,80,82]
AI algorithms, n (%)		
Convolutional neural networks	12 (20.0)	[35,47,48,56,62,69,72,78,83-86]
Feed-forward neural networks	12 (20.0)	[38,44,46,49,52,54,55,58,59,61,64,73]
Tree-based ensemble models	9 (15.0)	[34,36,60,65,68,75,79,81,90]
Support vector machines	6 (10.0)	[31,40,50,57,66,70]
Random forests	6 (10.0)	[37,41,42,76,80,87]
Recurrent neural networks	5 (8.3)	[63,71,74,82,89]
Others	10 (16.7)	[32,33,39,43,45,51,53,67,77,88]
Number of features		
Value, mean (SD; range)	23.3 (29.6; 3.0‐133.0)	[31-34,36,38,39,41,44,45,49-51,53,55,57-61,64-66,70,73,76,77,79-81,83,84,87,90]
Not reported, n (%)	26 (43.3)	[35,37,40,42,43,46-48,52,54,56,62,63,67-69,71,72,74,75,78,82,85,86,88,89]
Modal type, n (%)		
Unimodal	36 (60.0)	[33,35,37,39,40,43-46,49-59,63,65-67,70,73,74,76-80,82,85,86,90]
Multimodal	24 (40.0)	[31,32,34,36,38,41,42,47,48,60-62,64,68,69,71,72,75,81,83,84,87-89]
Unimodal input signal, n (%)		
Clinical information	19 (52.8)	[33,44,45,49-51,53-55,58,59,65-67,70,76,77,79,90]
Acoustic data	10 (27.8)	[37,39,40,43,46,52,57,74,78,82]
Physiological signals	6 (16.7)	[35,63,73,80,85,86]
Imaging data	1 (2.8)	[56]
Model validation method, n (%)		
Internal validation	41 (68.3)	[31-34,37-45,47,48,50,52-59,61,62,64,68,70,71,73-81,86,87]
External validation	18 (30.0)	[35,36,46,49,51,60,63,65,66,69,72,82-85,88-90]
Not reported	1 (1.7)	[67]
Data sources, n (%)		
Hospital	42 (70.0)	[31,34,36,38-48,50,52-58,62,64,67-69,74-79,81-83,85-90]
Community	16 (26.7)	[32,33,35,37,49,51,59-61,63,65,66,70-73]
Mixed community and hospital	1 (1.7)	[84]
Not reported	1 (1.7)	[80]
Data acquisition modality, n (%)		
PSG^a^-derived inputs	23 (38.3)	[31,32,34,35,38,41,54,60-64,69,71-73,80,84-89]
Non-PSG–derived inputs	37 (61.7)	[33,36,37,39,40,42-53,55-59,65-68,70,74-79,81-83,90]
Data accessibility, n (%)		
Proprietary datasets	44 (73.3)	[31-34,36,37,40-50,52-62,64,67-69,75-79,81,83-88]
Publicly available datasets	14 (23.3)	[35,38,39,51,63,65,66,70-74,80,89]
Mixed open and closed data	2 (3.3)	[82,90]

a PSG: polysomnography.

Unimodal inputs were used in 36 out of 60 (60%) studies [33,35,37,39,40,43-46,49-59,63,65-67,70,73,74,76-80,82,85,86,90], comprising clinical information (19/36, 53%) [33,44,45,49-51,53-55,58,59,65-67,70,76,77,79,90], acoustic data (10/36, 28%) [37,39,40,43,46,52,57,74,78,82], physiological signals (6/36, 17%) [35,63,73,80,85,86], and imaging data (1/36, 3%). Multimodal inputs were used in 24 out of 60 (40%) studies [31,32,34,36,38,41,42,47,48,60-62,64,68,69,71,72,75,81,83,84,87-89]. Internal validation was the predominant validation strategy (41/60, 68%) [31-34,37-45,47,48,50,52-59,61,62,64,68,70,71,73-81,86,87], whereas external validation was reported in 18 out of 60 (30%) studies [35,36,46,49,51,60,63,65,66,69,72,82-85,88-90]. The characteristics of each included study are listed in Table S2 in Multimedia Appendix 1.

### Quality Assessment and GRADE Certainty

**Figure 2.**
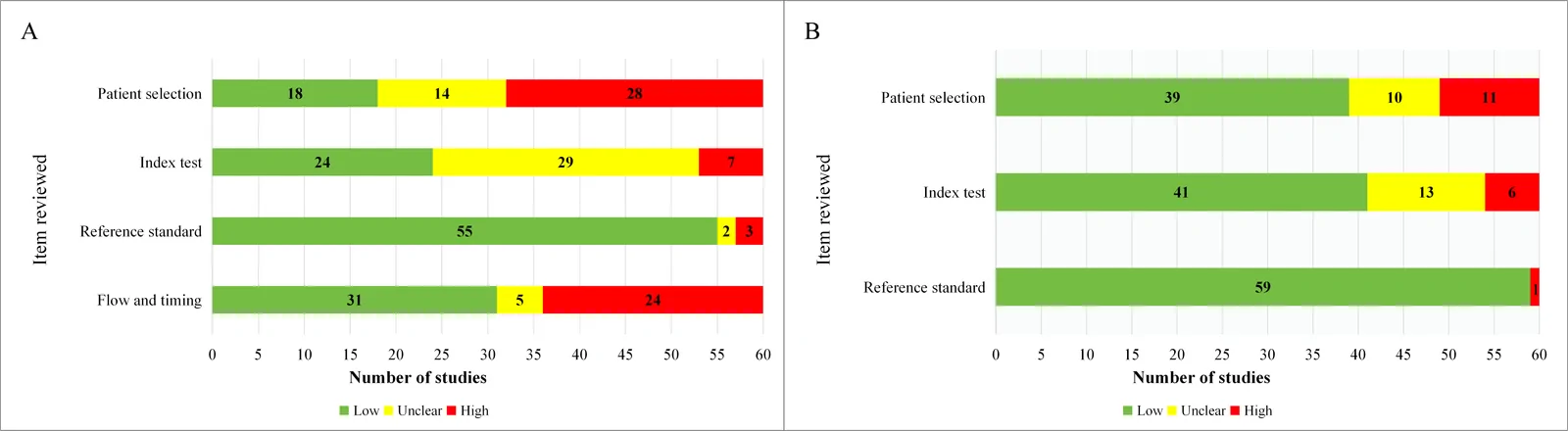
Summary of risk of bias and applicability concerns among the 60 [31-90] included studies. (A) Risk-of-bias judgments. (B) Applicability concerns.

Risk of bias and applicability concerns of the included studies were assessed using QUADAS-2, and the summary results are shown in Figure 2, with detailed judgments provided in Table S3 in Multimedia Appendix 1. In the risk-of-bias assessment, patient selection was rated as high or unclear in most studies (42/60, 70%) [31,34,36,38-48,50,52-58,62,64,67-69,74-79,81-83,85-90], mainly because many studies did not clearly report whether consecutive, random, or representative sampling was used. High or unclear risk of bias was also common in the index test domain, whereas the reference standard domain was generally rated as low risk, reflecting the use of PSG as the reference standard across included studies. For applicability concerns, most studies showed low concern across domains, particularly for the reference standard.

Table 2 summarizes the GRADE certainty assessment by input type and AHI threshold. For non-PSG–derived tools, the certainty of evidence was rated as very low across all three AHI thresholds. This was driven by serious risk-of-bias concerns, serious to very serious inconsistency, and serious concerns regarding publication bias. For PSG-derived tools, the certainty of evidence was rated as low across all 3 AHI thresholds, with downgrading due to serious risk-of-bias concerns and serious inconsistency. Indirectness and imprecision were not rated as serious concerns in either input group.

**Table 2.** GRADE (Grading of Recommendations Assessment, Development, and Evaluation) certainty assessment for AI-based obstructive sleep apnea (OSA) screening tools by input type and apnea-hypopnea index (AHI) threshold.

Groups	Risk of bias^a^	Inconsistency^b^	Indirectness^c^	Imprecision^d^	Publication bias^e^	Certainty
Non-PSG^f^–derived (events/h)						
AHI ≥5	Serious	Very serious	Not serious	Not serious	Serious	Very low
AHI ≥15	Serious	Serious	Not serious	Not serious	Serious	Very low
AHI ≥30	Serious	Very serious	Not serious	Not serious	Serious	Very low
PSG-derived (events/h)						
AHI ≥5	Serious	Serious	Not serious	Not serious	Not serious	Low
AHI ≥15	Serious	Serious	Not serious	Not serious	Not serious	Low
AHI ≥30	Serious	Serious	Not serious	Not serious	Not serious	Low

a Risk of bias was judged using the QUADAS-2 (Quality Assessment of Diagnostic Accuracy Studies 2) assessment. Evidence was rated down when concerns were present across studies or when high-risk domains were considered likely to affect the pooled diagnostic estimates.

b Inconsistency was judged using between-study heterogeneity, forest plots, and 95% prediction intervals (PIs). Evidence was rated down by one level for serious inconsistency and by two levels for very serious inconsistency when PIs suggested that diagnostic performance could vary substantially across future comparable settings.

c Indirectness was judged according to the applicability of the population, index test, reference standard, and AHI threshold to the review question.

d Imprecision was judged using the 95% CIs of pooled sensitivity and specificity and their implications for clinical interpretation.

e Publication bias was assessed by considering potential small-study effects, where applicable.

f PSG: polysomnography.

### Results of the Studies

#### Overview of Diagnostic Performance

Table 3 presents the pooled diagnostic performance of AI-based OSA screening tools across AHI thresholds and input sources. In the overall analysis, 31 threshold-specific datasets with 27,449 participants contributed data at AHI ≥5 events/hour, 37 datasets with 36,790 participants contributed data at AHI ≥15 events/hour, and 29 datasets with 27,824 participants contributed data at AHI ≥30 events/hour. At AHI ≥5 events/hour, the pooled sensitivity and specificity were 0.94 (95% CI 0.92‐0.96) and 0.77 (95% CI 0.69‐0.84), respectively; at AHI ≥15 events/hour, they were 0.87 (95% CI 0.84‐0.89) and 0.81 (95% CI 0.75‐0.85), respectively; and at AHI ≥30 events/hour, they were 0.83 (95% CI 0.79‐0.87) and 0.91 (95% CI 0.87‐0.94), respectively. Substantial heterogeneity was observed across thresholds, with *I*² values ranging from 91.8% to 98.9% for sensitivity and from 94.2% to 99.0% for specificity. The corresponding 95% PIs are reported in Table 3.

**Table 3.** Pooled diagnostic performance of AI-based screening tools for obstructive sleep apnea (OSA) across apnea-hypopnea index (AHI) thresholds and input types.

Groups	Total, n	Sample size, n	Pooled SE (95% CI); 95% PI		*I*² (%)	Pooled SD (95% CI); 95% PI		*I*² (%)	DOR^a^ (95% CI)	AUC^b^
Overall (events/h)										
AHI ≥5	31	27,449	0.94 (0.92‐0.96); 0.71‐0.99		98.9	0.77 (0.69‐0.84); 0.30‐0.96		94.2	56.48 (32.34‐98.66)	0.943
AHI ≥15	37	36,790	0.87 (0.84‐0.89); 0.66‐0.96		96.1	0.81 (0.75‐0.85); 0.39‐0.96		98.9	27.92 (18.32‐42.55)	0.907
AHI ≥30	29	27,824	0.83 (0.79‐0.87); 0.61‐0.94		91.8	0.91 (0.87‐0.94); 0.55‐0.99		99	53.23 (30.85‐91.84)	0.920
Non-PSG^c^–derived (events/h)										
AHI ≥5	14	10,528	0.92 (0.86‐0.96); 0.59‐0.99		96.4	0.70 (0.55‐0.81); 0.20‐0.96		93.6	26.74 (13.82‐51.74)	0.907
AHI ≥15	22	20,378	0.85 (0.81‐0.88); 0.64‐0.94		95.7	0.74 (0.67‐0.81); 0.36‐0.94		97.1	15.65 (10.13‐24.19)	0.871
AHI ≥30	16	11,806	0.81 (0.75‐0.86); 0.54‐0.94		91.2	0.85 (0.77‐0.90); 0.48‐0.97		93.3	24.28 (12.84‐45.92)	0.892
PSG-derived (events/h)										
AHI ≥5	17	16,921	0.96 (0.93‐0.97); 0.81‐0.99		91.5	0.82 (0.72‐0.89); 0.40‐0.97		94.5	102.53 (46.36–226.76)	0.962
AHI ≥15	15	16,412	0.90 (0.86‐0.93); 0.71‐0.97		93.2	0.88 (0.81‐0.92); 0.56‐0.98		96.9	63.40 (35.24‐114.08)	0.943
AHI ≥30	13	16,018	0.85 (0.81‐0.89); 0.68‐0.94		90.3	0.96 (0.93‐0.97); 0.84‐0.99		94.9	133.12 (76.30‐232.28)	0.957

a DOR: diagnostic odds ratio.

b AUC: area under the receiver operating characteristic curve.

c PSG: polysomnography.

#### Non-PSG–Derived Inputs

**Figure 3.**
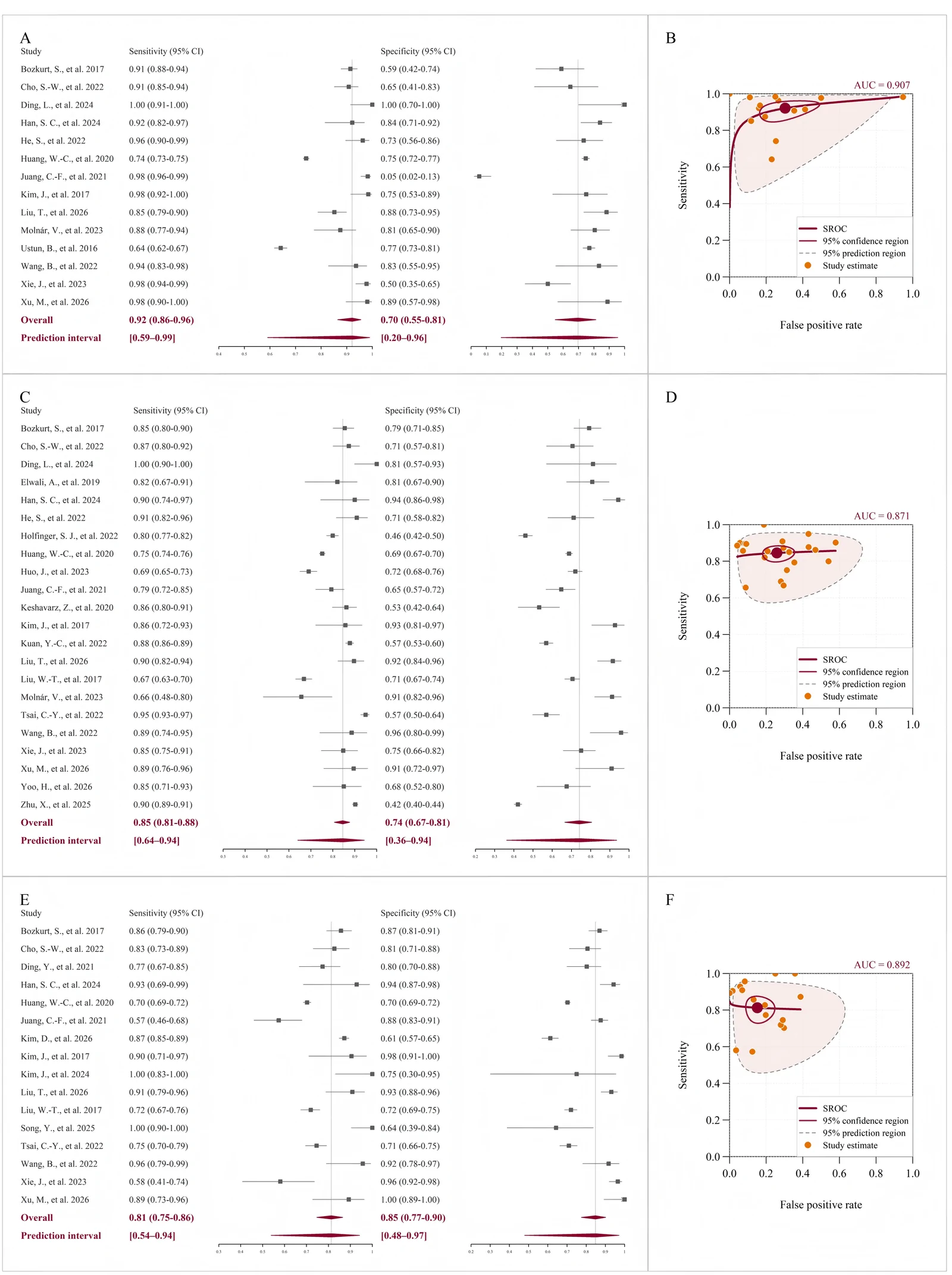
Screening performance of AI-based obstructive sleep apnea (OSA) tools using non-PSG–derived inputs. (A) Sensitivity and specificity forest plots at apnea-hypopnea index (AHI) ≥5 events/hour, (B) summary receiver operating characteristic (SROC) curve at AHI ≥5 events/hour, (C) sensitivity and specificity forest plots at AHI ≥15 events/hour, (D) SROC curve at AHI ≥15 events/hour, (E) sensitivity and specificity forest plots at AHI ≥30 events/hour, and (F) SROC curve at AHI ≥30 events/hour [33,37,39,40,42,46,48-51,53,55-59,65-67,74,76-78,81-83,90]. AUC: area under the receiver operating characteristic curve; PSG: polysomnography.

For non-PSG–derived inputs, at AHI≥5 events/hour, pooled sensitivity and specificity were 0.92 (95% CI 0.86‐0.96) and 0.70 (95% CI 0.55‐0.81), respectively; at AHI≥15 events/hour, they were 0.85 (95% CI 0.81‐0.88) and 0.74 (95% CI 0.67‐0.81), respectively; and at AHI≥30 events/hour, they were 0.81 (95% CI 0.75‐0.86) and 0.85 (95% CI 0.77‐0.90), respectively. The corresponding AUCs were 0.907, 0.871, and 0.892, respectively (Figure 3).

#### PSG-Derived Inputs

**Figure 4.**
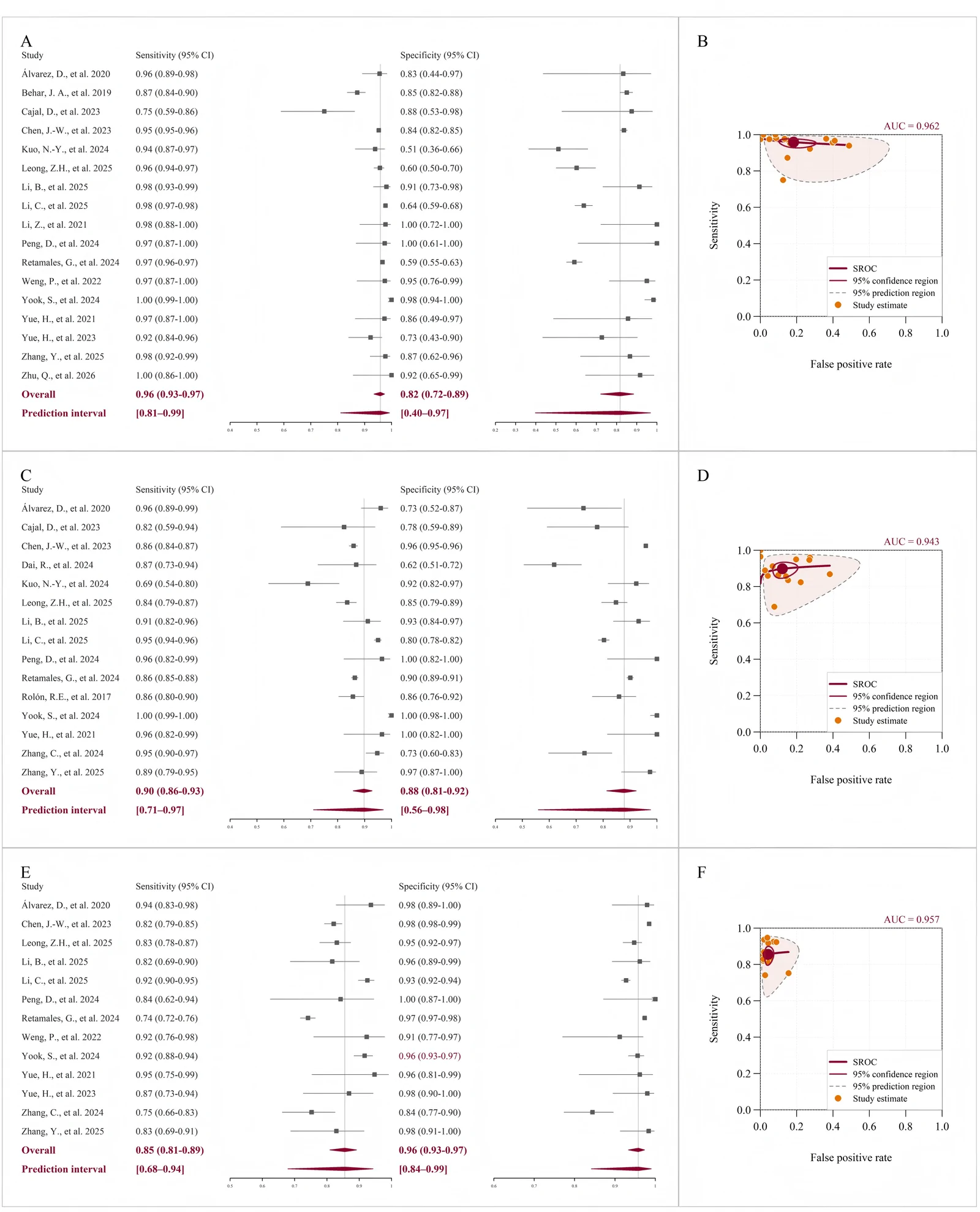
Screening performance of AI-based obstructive sleep apnea (OSA) tools using polysomnography (PSG)-derived inputs. (A) Sensitivity and specificity forest plots at apnea-hypopnea index (AHI) ≥5 events/hour, (B) summary receiver operating characteristic (SROC) curve at AHI ≥5 events/hour, (C) sensitivity and specificity forest plots at AHI ≥15 events/hour, (D) SROC curve at AHI ≥15 events/hour, (E) sensitivity and specificity forest plots at AHI ≥30 events/hour, and (F) SROC curve at AHI ≥30 events/hour [31,32,34,35,38,60-64,69,72,73,80,84-89].

For PSG-derived inputs, at AHI ≥5 events/hour, pooled sensitivity and specificity were 0.96 (95% CI 0.93‐0.97) and 0.82 (95% CI 0.72‐0.89), respectively; at AHI ≥15 events/hour, they were 0.90 (95% CI 0.86‐0.93) and 0.88 (95% CI 0.81‐0.92), respectively; and at AHI ≥30 events/hour, they were 0.85 (95% CI 0.81‐0.89) and 0.96 (95% CI 0.93‐0.97), respectively. The corresponding AUCs were 0.962, 0.943, and 0.957, respectively (Figure 4).

### Exploratory Subgroup Analyses

Exploratory subgroup analyses suggested possible variation in diagnostic performance across selected study and model characteristics. Among models using non-PSG–derived inputs, pooled sensitivity differed by region at AHI ≥15 events/hour (*P*=.02) and by algorithmic framework at AHI ≥15 events/hour (*P*<.001) and AHI ≥30 events/hour (*P*<.001). Among PSG-derived models, pooled sensitivity differed by algorithmic framework at AHI ≥5 events/hour (*P*<.001) and by validation method at AHI ≥5 events/hour (*P*=.002). Pooled specificity differed by data source at AHI ≥5 events/hour (*P*=.02), by algorithmic framework at AHI ≥15 events/hour (*P=*.003), and by validation method at AHI ≥15 events/hour (*P*=.003).

For PSG-derived models, externally validated models had higher pooled sensitivity at an AHI ≥5 events/hour than internally validated models, with estimates of 0.97 (95% CI 0.96‐0.98) and 0.93 (95% CI 0.87‐0.95), respectively. At an AHI ≥15 events/hour, externally validated models had higher pooled specificity than internally validated models, with estimates of 0.95 (95% CI 0.88‐0.98) and 0.79 (95% CI 0.70‐0.86), respectively. Detailed subgroup analysis results are shown in Tables S4 and S5 in Multimedia Appendix 1.

### Sensitivity Analyses and Small-Study Effects

Deeks’ funnel-plot asymmetry tests suggested potential small-study effects among non-PSG–derived models across AHI thresholds, whereas no clear funnel-plot asymmetry was observed for PSG-derived models. Although the aggregate sample size was large, the number of studies contributing to some threshold-specific and subgroup analyses was limited. Therefore, these findings should be interpreted as suggestive evidence of funnel plot asymmetry rather than definitive evidence of reporting bias or publication bias.

Leave-one-out sensitivity analyses demonstrated that the pooled sensitivity and specificity estimates were not driven by any single study across AHI thresholds. Although one study [53] exerted a relatively greater influence, its exclusion did not materially change the pooled results.

## Discussion

### Principal Findings

In this systematic review and meta-analysis, we evaluated the diagnostic accuracy of AI-based OSA screening tools across 3 clinically meaningful AHI thresholds and stratified the models by input source to distinguish non-PSG–derived tools from those using PSG-recorded channels. Overall, the included models showed high pooled sensitivity across the evaluated thresholds, supporting their potential role in identifying individuals who may require further sleep evaluation. Specificity was generally higher at more severe AHI thresholds, suggesting that screening performance may differ according to OSA severity. These patterns should be interpreted descriptively, and the clinical meaning of screening results should be considered in relation to disease prevalence, pretest probability, and the intended care setting [91]. Substantial heterogeneity and wide PIs were also observed, indicating that diagnostic performance may vary across study populations, input sources, algorithmic frameworks, and validation strategies [92]. Accordingly, the pooled estimates should be interpreted as summary measures of screening-oriented diagnostic accuracy across diverse study settings rather than as precise performance estimates for any specific clinical population or implementation context [92,93].

The input source was an important dimension for clinical interpretation. Non-PSG–derived tools use data that can be obtained before formal sleep testing and are therefore more relevant to front-end screening and pretest triage [13]. In this subgroup, pooled estimates showed favorable sensitivity and specificity across AHI thresholds. At the lower AHI threshold, the profile of higher sensitivity and comparatively lower specificity aligns with a broad case-identification role, whereas the higher specificity observed at more severe thresholds supports potential use in referral prioritization and severity-oriented risk stratification. PSG-derived models use signals collected during sleep testing and are therefore more closely aligned with screening-oriented classification or automated signal interpretation within sleep-testing pathways [13]. These models also showed favorable screening performance, particularly higher specificity at moderate-to-severe and severe OSA thresholds. Because this analysis was based on stratified pooled estimates rather than direct comparisons, differences between input-source groups should be interpreted cautiously.

Exploratory subgroup analyses suggested that screening-oriented diagnostic accuracy varied across selected study and model characteristics. Sensitivity varied by region and algorithmic framework among non-PSG–derived models and by algorithmic framework and validation method among PSG-derived models. Specificity also varied by data source, algorithmic framework, and validation method. In interpreting these results, the 95% CIs should be understood as reflecting the precision of the pooled average estimates, whereas the 95% PIs describe the expected distribution of diagnostic performance across future comparable studies or implementation settings. The wide PIs indicate that performance in such settings may differ meaningfully from the pooled summary estimates [93]. Because these subgroup analyses were exploratory, these findings should be regarded as hypothesis-generating rather than confirmatory.

Overall, these findings support the potential value of AI-based tools for OSA screening and severity-oriented risk stratification, while emphasizing that substantial heterogeneity, wide PIs, risk-of-bias concerns, and low or very low certainty of evidence according to GRADE temper the generalizability of the pooled estimates.

### Research and Practical Implications

Consistent with previous reviews, these findings suggest that AI-based approaches may support OSA risk stratification by extracting clinically relevant information from diverse data sources, including demographic, physiological, acoustic, imaging, and multimodal data [20,22,94]. The clinical implications of AI-based OSA screening tools vary across the OSA severity spectrum and by model input source [95]. At AHI ≥5 events/hour, these tools may be most useful for broad case identification, where high sensitivity helps reduce missed cases. At AHI ≥15 events/hour, screening has greater relevance for diagnostic referral and treatment planning. At AHI ≥30 events/hour, improved specificity may help prioritize individuals who require timely diagnostic testing or specialist care [96], particularly when diagnostic resources are limited. Input sources also shape practical use. Non-PSG–derived tools use data available before formal sleep testing, such as clinical variables, questionnaires, wearable sensor data [22,23], acoustic signals, or home-based physiological measures. These tools are most relevant for front-end screening and pretest triage [21,94]. Their practical value lies in translating information that is already available before PSG or HSAT into a structured estimate of OSA risk, thereby supporting earlier recognition of individuals who may benefit from further sleep evaluation and helping standardize referral decisions across clinical settings [13,22]. By contrast, PSG-derived models have a different practical role. Because they use signals collected during sleep testing, they are more relevant to automated sleep-signal interpretation, reduced-channel assessment, and workflow support within sleep centers [97]. These tools may help reduce the manual scoring burden and improve efficiency after patients have entered the sleep-testing pathway [98]. Traditional questionnaire-based tools, such as STOP-Bang, remain clinically useful because they are simple, inexpensive, and easy to implement [16,99]. AI-based models may offer a more flexible approach by integrating heterogeneous data sources, but direct comparisons with established screening questionnaires remain limited and should be prioritized in future studies.

Taken together, the distinguishing contribution of this review lies in organizing the evidence according to 2 clinically relevant dimensions: disease-severity threshold and model input source. Unlike many previous narrative, scoping, or modality-specific reviews that have described AI applications in OSA more broadly [21,22,94,97,100], this review separately evaluates diagnostic performance at AHI thresholds of ≥5, ≥15, and ≥30 events/hour and distinguishes non-PSG–derived screening models from PSG-derived models. This approach contributes to the field by providing a more clinically interpretable framework for comparing AI-based OSA screening tools and for relating model performance to intended use. From a practical perspective, these findings may provide preliminary evidence for considering how different AI-based screening tools could be positioned within OSA care, including early screening, referral triage, reduced-channel assessment, and sleep-laboratory workflow support [22,94,100]. Given the heterogeneity of existing studies, limited external validation, and low or very low certainty of evidence, these tools are best viewed as complementary to established screening approaches [21,100,101].

### Strengths and Limitations

This review has several methodological strengths. First, the analyses were structured around prespecified clinically relevant AHI thresholds and input-source categories, which reduced clinical ambiguity and improved the interpretability of the pooled estimates. Second, diagnostic accuracy was synthesized using bivariate random-effects models, allowing sensitivity and specificity to be jointly estimated while accounting for between-study heterogeneity [93,102]. Third, risk of bias and applicability concerns were systematically assessed using QUADAS-2 [27], and the certainty of evidence was evaluated using GRADE [28], providing a structured basis for interpreting the pooled diagnostic estimates in light of methodological quality, applicability, inconsistency, imprecision, and potential publication bias.

Several limitations should be acknowledged. First, substantial heterogeneity was observed across AHI thresholds and input categories, likely reflecting differences in study populations, reference scoring rules, input modalities, algorithms, validation strategies, and reporting quality [94,97,100]. Therefore, pooled estimates should be interpreted as average performance across heterogeneous settings rather than as expected performance in any single clinical context [102]. Second, many included studies used retrospective, internally validated, hospital-derived, or clinically referred samples, which may limit generalizability to primary care, community-based screening, and large-scale population-level case identification [100,103]. Third, several subgroup analyses were limited by small numbers of studies, and potential small-study effects were observed among non-PSG–derived models, suggesting that these exploratory findings should be interpreted cautiously [104]. Fourth, PSG-derived channel models should not be interpreted as equivalent to non-PSG screening tools because they rely on data collected during sleep testing and therefore follow a different clinical implementation pathway [97,98]. Finally, because model decision cutoffs were inconsistently reported and threshold effects could not be formally assessed, variability in cutoff selection may have contributed to between-study heterogeneity in diagnostic accuracy [102,105]. Future research should prioritize prospective multicenter external validation across clinically relevant AHI thresholds, standardized reporting of model development and validation [106], calibration assessment [107], clinical utility analysis [108], and direct comparison with existing screening tools [16,99]. Evaluation across diverse clinical settings and patient subgroups is also needed to determine whether AI-based screening can improve referral efficiency, reduce diagnostic delays, and support equitable access to sleep care [22,94,101].

### Conclusions

In conclusion, AI-based tools showed generally favorable screening performance for OSA across clinically relevant AHI thresholds, although wide PIs indicate that performance may vary across future comparable populations and clinical settings. The methodological innovation of this review was the synthesis of diagnostic accuracy across 3 AHI thresholds while distinguishing non-PSG–derived from PSG-derived models. Compared with previous broad or modality-specific reviews, this pathway-specific approach links model performance to intended use and offers a more clinically interpretable basis for model comparison and future evaluation. The findings may help clarify potential roles for non-PSG–derived tools in front-end screening and referral prioritization and for PSG-derived models in reduced-channel assessment and sleep-laboratory workflow support. Given the substantial heterogeneity, limited external validation, and low or very low certainty of evidence, prospective validation in representative populations is needed before routine clinical implementation.

## Supplementary material

10.2196/92399 Multimedia Appendix 1 Supplementary tables and figures for the systematic review and meta-analysis.

10.2196/92399 Checklist 1 PRISMA checklists (PRISMA 2020, PRISMA-DTA, PRISMA-S).

## Abbreviations

AHI: apnea-hypopnea index
AUC: area under the receiver operating characteristic curve
DOR: diagnostic odds ratio
FN: false negative
FP: false positive
GRADE: Grading of Recommendations Assessment, Development, and Evaluation
HSAT: home sleep apnea testing
OSA: obstructive sleep apnea
PRISMA: Preferred Reporting Items for Systematic Reviews and Meta-Analyses
PSG: polysomnography
QUADAS-2: Quality Assessment of Diagnostic Accuracy Studies 2
RDI: respiratory disturbance index
SROC: summary receiver operating characteristic
TN: true negative
TP: true positive
