# Supplementary material for: Performance of AI-Based Screening Tools for Obstructive Sleep Apnea Across Apnea-Hypopnea Index Thresholds: Systematic Review and Meta-Analysis
Source: J Med Internet Res. 2026 Sep 11;28:e92399. doi: 10.2196/92399 (PMC13568598; doi:10.2196/92399)
Supplement: Supplementary file 1 [file jmir-v28-e92399-s001.docx]

**SUPPLEMENTARY MATERIALS**

**Performance of AI–Based Screening Tools for Obstructive Sleep Apnea Across Apnea–Hypopnea Index Thresholds: A Systematic Review and Meta-Analysis**

**________________________________________________________**

**This supplementary document supports the main manuscript and provides detailed methodological descriptions, additional results, comprehensive search strategies, and quality assessment data.**

**Table of Contents**

**Table S1.** Search strategies for each database...........................................................................................................................................................................................................3

**Table S2**. Characteristics of the included studies (N=60).........................................................................................................................................................................................5

**Table S3.** QUADAS-2 quality assessment of the included studies...........................................................................................................................................................................16

**Table S4.** Exploratory Subgroup Analysis of AI-Based OSA Screening Performance Using Non-PSG-Derived Inputs Across AHI Thresholds..................................................19

**Table S5.** Exploratory Subgroup Analysis of AI-Based OSA Screening Performance Using PSG-Derived Inputs Across AHI Thresholds..........................................................23

**Table S1.** Search strategies for each database.

| **Database** | **Search strategy** | **Filters and Limits** | **Number** |
| --- | --- | --- | --- |
| Pubmed | ("Sleep Apnea, Obstructive"[Mesh] OR "Sleep Apnea Syndromes"[Mesh] OR "obstructive sleep apnea"[tiab] OR "obstructive sleep apnoea"[tiab] OR "obstructive sleep apnea syndrome"[tiab] OR OSA[tiab] OR OSAS[tiab] OR OSAHS[tiab] OR "sleep-disordered breathing"[tiab] OR "sleep disordered breathing"[tiab]) AND ("Artificial Intelligence"[Mesh] OR "Machine Learning"[Mesh] OR "Neural Networks, Computer"[Mesh] OR artificial intelligence[tiab] OR machine learning[tiab] OR deep learning[tiab] OR neural network*[tiab] OR deep neural network*[tiab] OR convolutional neural network*[tiab] OR recurrent neural network*[tiab] OR support vector machine*[tiab] OR random forest*[tiab] OR gradient boosting[tiab] OR xgboost[tiab] OR ensemble learning[tiab] OR "computer vision"[tiab] OR transformer*[tiab] OR multimodal*[tiab])  AND ("Mass Screening"[Mesh] OR screen*[tiab] OR "diagnostic accuracy"[tiab] OR detect*[tiab] OR diagnos*[tiab] OR classif*[tiab] OR predict*[tiab] OR triag*[tiab]) | The database searches were limited to records published from January 1, 2016, to May 3, 2026.No language or publication type filters were applied. | 1,073 |
| Web of Science | TS=(("obstructive sleep apnea" OR "obstructive sleep apnoea" OR "obstructive sleep apnea syndrome" OR OSA OR OSAS OR OSAHS OR "sleep-disordered breathing" OR "sleep disordered breathing")  AND ("artificial intelligence" OR "machine learning" OR "deep learning" OR "neural network*" OR "deep neural network*" OR "convolutional neural network*" OR "recurrent neural network*" OR "support vector machine*" OR "random forest*" OR "gradient boosting" OR xgboost OR "ensemble learning" OR "computer vision" OR transformer* OR multimodal*)  AND (screen* OR "diagnostic accuracy" OR detect* OR diagnos* OR classif* OR predict* OR triag*)) | The database searches were limited to records published from January 1, 2016, to May 3, 2026. No language or publication type filters were applied. | 1,945 |
| Embase | ('obstructive sleep apnea'/exp OR 'sleep disordered breathing'/exp OR 'obstructive sleep apnea':ti,ab OR 'obstructive sleep apnoea':ti,ab OR 'obstructive sleep apnea syndrome':ti,ab OR OSA:ti,ab OR OSAS:ti,ab OR OSAHS:ti,ab OR 'sleep-disordered breathing':ti,ab OR 'sleep disordered breathing':ti,ab)  AND ('artificial intelligence'/exp OR 'machine learning'/exp OR 'neural network'/exp OR 'deep learning'/exp OR 'artificial intelligence':ti,ab OR 'machine learning':ti,ab OR 'deep learning':ti,ab OR 'neural network*':ti,ab OR 'deep neural network*':ti,ab OR 'convolutional neural network*':ti,ab OR 'recurrent neural network*':ti,ab OR 'support vector machine*':ti,ab OR 'random forest*':ti,ab OR 'gradient boosting':ti,ab OR xgboost:ti,ab OR 'ensemble learning':ti,ab OR 'computer vision':ti,ab OR transformer*:ti,ab OR multimodal*:ti,ab)  AND ('mass screening'/exp OR screening:ti,ab OR 'diagnostic accuracy':ti,ab OR detect*:ti,ab OR diagnos*:ti,ab OR classif*:ti,ab OR predict*:ti,ab OR triag*:ti,ab) | The database searches were limited to records published from January 1, 2016, to May 3, 2026. No language or publication type filters were applied. | 2,378 |
| Scopus | TITLE-ABS-KEY(("obstructive sleep apnea" OR "obstructive sleep apnoea" OR "obstructive sleep apnea syndrome" OR OSA OR OSAS OR OSAHS OR "sleep-disordered breathing" OR "sleep disordered breathing")  AND ("artificial intelligence" OR "machine learning" OR "deep learning" OR "neural network*" OR "deep neural network*" OR "convolutional neural network*" OR "recurrent neural network*" OR "support vector machine*" OR "random forest*" OR "gradient boosting" OR xgboost OR "ensemble learning" OR "computer vision" OR transformer* OR multimodal*)  AND (screen* OR "diagnostic accuracy" OR detect* OR diagnos* OR classif* OR predict* OR triag*)) | The database searches were limited to records published from January 1, 2016, to May 3, 2026. No language or publication type filters were applied. | 2,281 |

Note: All database searches were conducted on May 3, 2026.

**Table S2:** Characteristics of the included studies (N = 60).

|  | Study ID | Country | N | Male proportion | Age | AI algorithms | Input types | No. of features | Validation method |
| --- | --- | --- | --- | --- | --- | --- | --- | --- | --- |
| 1 | Álvarez, D., et al. 2020 | Spain | 239 | 0.686 | 56 (46–65) | SVM | SpO₂ + nasal airflow | 18 | Train-test split |
| 2 | Behar, J. A., et al. 2019 | Brazil | 887 | 0.451 | 20–80; No OSA: 34 ± 17; Mild: 47 ± 18; Mod: 50 ± 20; Sev: 57 ± 16.5 | OxyDOSA^a^: LASSO + LR; LR-SpO₂; LR-ODI; LR-SB | PSG-derived SpO₂ + demographic variables | 12 | 5-fold CV |
| 3 | Bozkurt, S., et al. 2017 | Turkey | 338 | 0.754 | No OSA 44.8±10.1; OSA 49.2±11.0 | Bayesian Network^a^; DT; RF; NN; LR | Age + gender + BMI + NC + smoking + ESS + ENT physical examination | 14 | 10-fold CV |
| 4 | Cajal, D., et al. 2024 | Belgium | 94 | NR | 44.5 ± 11.4 | Bagged Trees | PSG-derived PPI, SpO₂ | 6 | Train-test split + 5-fold CV |
| 5 | Chen, J.-W., et al. 2023 | Taiwan | 14433 | NR | SHHS1: ≥40;  Overall: NR | DNN-CSPNet-SENet-ResNet^a^; DNN variants | PSG-derived SpO₂ | NR | External validation |
| 6 | Chen, Q., et al. 2023 | China | 653 | 0.772 | 50.3 ± 12.8 | CatBoost^a^; LightGBM; LR; SVM | 2D facial photograph + 19 clinical variables | 87 | External validation |
| 7 | Cho, S.-W., et al. 2022 | South Korea | 423 | 0.842 | 48.1±12.8 | RF^a^ | Smartphone-recorded sleep breathing sounds | NR | Train-test split + 10-fold CV |
| 8 | Dai, R., et al. 2024 | China | 571 | 0.799 | Mod-Sev: 44.1 ± 12.3; No OSA-Mild: 41.6 ± 13.1 | ANN^a^; LR; NB; SVM; RF; DT | Age + sex + BMI + sleep HR | 4 | Train-test split + 10-fold CV |
| 9 | Ding, L., et al. 2024 | China | 120 | 0.792 | 32–49 | MFCC-kNN + VGG16-kNN + PANN-kNN fusion^a^; variants | Snoring sounds | 36 | LOSOCV + Train-test split |
| 10 | Ding, Y., et al. 2021 | China | 151 | 1.000 | AHI≥ 30: 39.2 ± 8.7; AHI < 30: 39.7 ± 10.3;  AHI ≥ 10: 39.4 ± 8.9; AHI < 10: 39.7 ± 11.1 | SVM | Awake voice | NR | LOOCV |
| 11 | dos Santos, R. R., et al. 2025 | Brazil | 291 | 0.405 | No OSA: 41 (30–55); Mild: 52 (42–63); Mod: 56.5 (38.75–65); Sev: 56 (46–63) | RF | PSG-derived HRV, SpO₂ + anthropometric variables | 41 | 10-fold CV |
| 12 | Elwali, A., et al. 2019 | Canada | 199 | 0.533 | AHI < 15: 48.60 ± 12.69; AHI ≥ 15: 52.18 ± 11.55 | RF | Tracheal breath sounds + anthropometric variables | NR | Train-test split + OOB validation |
| 13 | Fang, L., et al. 2025 | China | 465 | 0.845 | 38.6 ± 10.2 | AST^a^; SVM; kNN; RF | Simulated snoring sounds | NR | Train-test split |
| 14 | Ge, S., et al. 2024 | China | 2064 | 0.789 | 54.91 ± 12.78; 51.43 ± 12.43 | MLP^a^; SVM; RF; LR; AdaBoost; GB; XGBoost | Clinical variables + laboratory variables + carotid plaque | 24 | Train-test split + 10-fold CV |
| 15 | Han, H., et al. 2023 | South Korea | 4014 | 0.708 | 53 (40–62) | Clustering + feature engineering^a^; LightGBM; XGBoost; CatBoost; RF | Structured clinical data | 33 | Train-test split + 5-fold CV |
| 16 | Han, S. C., et al. 2024 | South Korea | 101 | 0.495 | 48.3 ± 14.9 | DNN | Smartphone-recorded breath sounds | NR | External validation |
| 17 | Hanif, U., et al. 2021 | USA, Canada | 1366 | 0.470 | 45.9 ± 14.8 | CNN + MLPs | 3D facial scan + age + sex + modified STOP-Bang questionnaire | NR | 10-fold CV |
| 18 | He, S., et al. 2022 | China | 393 | 0.784 | 39.1 ± 12.0 | EfficientNet-B4 + LightGBM voting^a^; EfficientNet-B4 | Craniofacial photograph + age + sex + BMI + NC | NR | Train-test split |
| 19 | Holfinger, S., J., et al. 2022 | Multiple countries^b^ | 24660 | 0.491 | Retrospective SAGIC: ~50.8; Prospective SAGIC: 50.1 ± 14.1; SHHS: 63.3 ± 11.2 | ANN^a^; RF; kSVM | Age + sex + BMI + race | 4 | External validation |
| 20 | Huang, W.-C., et al. 2020 | Taiwan | 6875 | 0.760 | 47.8 ± 14.5 | SVM | Age + sex + anthropometric variables + comorbidities + sleep symptoms + ESS | 2/6/6 selected for AHI ≥ 5/15/30 | 5-fold CV |
| 21 | Huo, J., et al. 2022 | USA | 5243 | 0.512 | SHHS Train: 64.68 ± 11.29; SHHS Test: 60.18 ± 8.32; WSC: 56.42 ± 8.13 | BASH-GN^a^; LR subtype classifiers; SVC; kNN; DT; ET; AdaBoost; GNB; RF | Clinical questionnaire variables: age, sex, BMI, NC, snoring loudness, hypertension | 6 | External validation |
| 22 | Juang, C., et al. 2021 | Taiwan | 300 | 0.793 | 41.3 ± 13.2 | EFNN | Waist circumference + end-PSG BP + BP change | NR | 5-fold CV |
| 23 | Juang, C.-F., et al. 2023 | Taiwan | 1197 | 0.782 | 45.0 (35.0–57.0) | IFES^a^; EFNN; MLP; SVM; Stepwise regression | NC + waist circumference + waking BP | 3 | 5-fold CV |
| 24 | Kang, C., et al. 2023 | South Korea | 139 | 0.770 | Younger: 32.91 ± 8.9; Older: 52.47 ± 7.22 | MLP^a^; SVM; kNN | PSG-derived EEG | NR | 5-fold CV + Train-test split |
| 25 | Keshavarz, Z., et al. 2020 | Iran | 231 | 0.619 | ≥ 18 | NN^a^; Naive Bayes; LR; kNN; SVM; RF | Self-reported symptoms + BMI | 8 | 10-fold CV |
| 26 | Kim, D., et al. 2026 | South Korea | 1381 | 0.720 | AHI < 30: 46.82 ± 18.22;  AHI≥ 30: 51.17 ± 15.23 | MHCA-EfficientNet-B2^a^; MHSA; MHDA; CNN/Transformer baselines | Multi-view facial images: frontal + axial + lateral | NR | 10-fold CV |
| 27 | Kim, J., et al. 2017 | South Korea | 83 | 0.675 | 48.7 ± 17.5 | SVM | Respiratory sounds | 18 | LOOCV |
| 28 | Kim, J., et al. 2024 | USA | 66 | 0.682 | 52.4 ± 14.6 | DNN-PCA^a^; RF; AdaBoost; kNN; SVM; DT | Age + sex + questionnaire variables | 38 | Train-test split + 3-fold CV |
| 29 | Kuan, Y.-C., et al. 2022 | Taiwan | 9422 | 0.690 | 49.2±14.0 | ANN^a^; LR | Age + sex + BMI | 3 | Train-test split + 5-fold CV |
| 30 | Kuo, N.-Y., et al. 2024 | Taiwan, USA | 9673 | 0.487 | SHHS: 64.55 ± 11.17; TVGH Train: 52.35 ± 15.61; TVGH Test: 51.80 ± 15.16 | Sequential ExtraTrees + CatBoost^a^; RF; ExtraTrees | Questionnaire variables + PSQI + PSG-derived SpO₂ | 133 | External validation |
| 31 | Leong, Z.H., et al. 2025 | Singapore | 2996 | 0.672 | 47.0±14.2 | NN^a^; RF; SVM; GB; AdaBoost; kNN; LR | PSG-derived oximetry: ODI, SpO₂ nadir, time SpO₂<90%, time SpO₂<85% + age, gender, NC, BMI, ESS | 7 | Train-test split + 10-fold CV |
| 32 | Li, B., et al. 2025 | China | 426 | NR | NR | DFNet^a^; ResNet; MLSTM-FCN; Omni-scale CNNs; InceptionTime; XCM; XceptionTime | Overnight snoring audio + age + BMI + medical history | NR | Train-test split |
| 33 | Li, C., et al. 2025 | USA | 8444 | NR | ≥ 40 | MSC-Mamba-LSTM^a^; MCA attention | PSG-derived SpO₂ | NR | External validation |
| 34 | Li, Z., et al. 2021 | China | 181 | 0.740 | No OSA: 34.1 ±1 1.2; OSA: 41.5 ± 10.6 | FNN^a^; LR; RUSBoosted Trees; Complex Tree; linear SVM; linear discriminant | PSG-derived ECG, SpO₂ + BMI | 10 | Train-test split |
| 35 | Liu, T., et al. 2026 | China | 4036 | 0.608 | No OSA 41.8±12.4; Mild 46.2±11.9; Moderate 50.7±11.2; Severe 54.3±11.8 | XGBoost^a^; SVM; ANN; LR | Modified ESS items + snoring + tiredness + witnessed apneas + hypertension + age + sex + BMI + NC | 15 | External validation |
| 36 | Liu, W.-T., et al. 2017 | Taiwan | 6399 | 0.753 | TMUH male 45.3±12.8; TMUH female 45.4±14.0; SHH male 46.6±12.9; SHH female 49.5±13.8 | SVM | Age+ BMI+ NC+ waist circumference | 4 | External validation |
| 37 | Molnár, V., et al. 2023 | Hungary | 100 | 0.740 | 42.2 | FDA^a^; LDA; QDA; NN | Age + sex + BMI + NC + Berlin questionnaire + ESS | NR | NR |
| 38 | Monna, F., et al. 2022 | France | 267 | 1.000 | 59 ± 9 | XGBoost^a^; LR; LDA; kNN; SVM; RF; ANN; AdaBoost; ExtraTrees; NB | 3D craniofacial scan + anthropometric variables + questionnaire/symptom data | NR | Nested 4-fold CV |
| 39 | Peng, D., et al. 2024 | China | 450 | NR | FAH: 30–48; CMH: 32–48 | Feature-fusion CNN^a^; 1D CNN; Mr-ResNet; LSTM-CNN; kNN; SVM; 6-layer CNN | PSG-derived nasal pressure airflow, SpO₂ | NR | External validation |
| 40 | Ramesh, J., et al. 2021 | USA | 1479 | 0.532 | 58.20 ± 8.04 | SVM^a^; XGBoost; LightGBM; CatBoost; RF; kNN; LR | EHR clinical variables + anthropometric variables + sleep history/questionnaire-derived symptoms | 8 | 5-fold CV + Train-test split |
| 41 | Ramesh, J., et al. 2025 | USA | 2564 | NR | NR | CNN-BiLSTM-Attention^a^ | PSG-derived hypnodensity/hypnogram images + sleep features + EHR/medical history | NR | Train-test split |
| 42 | Retamales, G., et al. 2024 | USA | 10643 | NR | SHHS: 64.6 (39–90); MrOS: 77.5 (67–90); MESA: 69.4 (54–94) | CNN + LightGBM^a^ | Abdominal movement + SpO₂; thoracic movement tested | NR | External validation |
| 43 | Rolón, R.E., et al. 2017 | USA | 954 | NR | NR | MDCS-OD + MLP^a^; FULL-OD; MDAS-OD; FULL-CD; MDAS-CD; MDCS-CD | PSG-derived SpO₂ / pulse oximetry | 64 | Train-test split |
| 44 | Song, Y., et al. 2025 | Greece | 305 | NR | NR | ConvNeXt + LSTM | Non-contact overnight sleep respiratory sounds | NR | Train-test split |
| 45 | Su, Z., et al. 2023 | USA | 280 | NR | ≥ 18; detailed age: NR | DeepXGBoost with XGBoost clinical variables^a^; DeepXGBoost; XGBoost; EfficientNet-OSA | 2D frontal craniofacial images + clinical variables | NR | 5-fold CV |
| 46 | Tsai, C.-Y., et al. 2022 | Taiwan | 3503 | 0.663 | No OSA-Mild: 43.19 ± 13.85; Mod: 49.48 ± 13.26; Sev: 49.85 ± 13.04 | RF^a^; LR; SVM; XGBoost; kNN; NB | Anthropometric measurements + body composition | 28 | Train-test split + 10-fold CV |
| 47 | Ustun, B., et al. 2016 | USA | 1922 | 0.587 | No OSA: 44 (32–53); OSA: 53 (43–63) | SLIM^a^; LASSO; Ridge; Elastic Net; C5.0; SVM | Age + sex + BMI + comorbidities + smoking | 10 | 10-fold CV |
| 48 | Wang, B., et al. 2022 | China | 194 | 0.758 | Train: 40.7 ± 10.8; Val: 40.2 ± 9.3;  Test: 41.6 ± 11.1 | OSAnet^a^; deep CNN object-detection model | Sleep sounds | NR | Train-test split |
| 49 | Wang, K.-J., et al. 2016 | Taiwan | 3345 | NR | No OSA: 36.73 ± 14.54; Mild: 45.95 ± 28.50; Mod: 46.76 ± 13.20; Sev: 47.46 ± 18.62 | SMOTE + FDT^a^; FDT; LR; BPN; LVQ; SVM; C4.5 | Anthropometric variables + questionnaire variables | 8 | 10-fold CV |
| 50 | Weng, P., et al. 2022 | China | 60 | NR | NR | RF^a^; SVM; kNN; GNB | Single-lead ECG-derived HRV | 4 | 2-fold CV |
| 51 | Xie, J., et al. 2023 | Netherlands | 172 | 0.634 | 50.3 ± 14.9; range: 18–86 | XGBoost^a^ | Full-night snoring audio + age + BMI | 93 | 5-fold CV |
| 52 | Xu, M., et al. 2026 | Greece, China | 346 | NR | ≥ 18 | Dual-Stream CRNN^a^ | Sleep audio | NR | External validation |
| 53 | Yoo, H., et al. 2026 | South Korea | 710 | 0.765 | No OSA: 40.68 ± 13.34;  Mild: 45.02 ± 15.33; Mod: 51.79 ± 13.42; Sev: 52.52 ± 12.08 | Trimodal ShuffleNet-V2 + DNN^a^; ShuffleNet-V2; DNN; EfficientNet-B7; WideResNet50-2; InceptionV3; ViT-B/16 | 2D facial photograph + cephalometric radiograph + clinical variables | 7 | External validation |
| 54 | Yook, S., et al. 2024 | South Korea, USA | 6421 | 0.513 | SMC HS:63.2±12.27;  SMC OSA:74.6±13.49;  SHHS1 HS:59.7±11.08; SHHS1 Mild:63.5±11.13; SHHS1 Mod:65.3±10.6; SHHS1 Sev:65.6±10.79 | Xception Network + SVM^a^; 1D CNN | PSG-derived respiratory flow, SpO₂, ECG + age + sex + BMI | 6 | External validation |
| 55 | Yue, H., et al. 2021 | China | 450 | 0.878 | FAH: 40.0 (31.0–48.0); CMH: 39.0 (32.0–48.0) | OSASS / Mr-ResNet | PSG-derived single-channel nasal pressure airflow | NR | External validation |
| 56 | Yue, H., et al. 2023 | China | 436 | 0.878 | 39.0 (31.0–48.0) | SE-MSCNNa; TW-MLP; Modified LeNet-5; CNN-LSTM | PSG-derived ECG | NR | Train-test split |
| 57 | Zhang, C., et al. 2024 | China | 798 | 0.799 | 43.51 ± 12.77 | RF^a^; LR | PSG-derived ECG-HRV + clinical/demographic variables + BP + lowest SpO₂ | 13 | Train-test split + 10-fold CV |
| 58 | Zhang, Y., et al. 2025 | China | 510 | 0.747 | 18–65;  Train: 37.51 ± 12.78;  Val: 37.51 ± 11.46; Test: 36.90 ± 12.68 | Multimodal multiscale Transformer^a^; LKCNN; 1DSE; BiGRU; MM-Transformer | PSG-derived ECG, SpO₂ | NR | External validation |
| 59 | Zhu, Q., et al. 2026 | NR | 95 | 0.821 | Apnea-ECG: 45.1 ± 10.8; UCD: 50 ± 10 | LSTM-SVM fusion^a^; RNN-SVM; CNN-SVM; LSTM; RNN; CNN | ECG + biometric variables | NR | External validation |
| 60 | Zhu, X., et al. 2025 | China, USA | 13016 | 0.686 | D1: 43.27 ± 12.53; D2: 42.50 ± 11.71; D3: 63.36 ± 10.66 | LightGBM^a^; XGBoost; RF; FNN; CNN | Age + sex + multidimensional obesity indicators | 21 | External validation |

^a^Final or main model extracted for analysis.

^b^Multiple countries refers to the United States, Australia, Taiwan, Iceland, Germany, and China.

Abbreviations: AHI, apnea-hypopnea index; ANN, artificial neural network; BMI, body mass index; BP, blood pressure; CNN, convolutional neural network; CRNN, convolutional recurrent neural network; CV, cross-validation; DNN, deep neural network; DT, decision tree; ECG, electrocardiography; EEG, electroencephalography; EHR, electronic health record; ENT, ear, nose, and throat; ESS, Epworth Sleepiness Scale; FNN, feed-forward neural network; GB, gradient boosting; GNB, Gaussian naïve Bayes; HR, heart rate; HRV, heart rate variability; kNN, k-nearest neighbors; LASSO, least absolute shrinkage and selection operator; LightGBM, light gradient boosting machine; LOOCV, leave-one-out cross-validation; LOSOCV, leave-one-subject-out cross-validation; LR, logistic regression; LSTM, long short-term memory; MFCC, Mel-frequency cepstral coefficients; MLP, multilayer perceptron; Mod, moderate OSA; Mod-Sev, moderate-to-severe OSA; NB, naïve Bayes; NC, neck circumference; NN, neural network; No OSA, no obstructive sleep apnea; No OSA-Mild, no OSA or mild OSA; NR, not reported; OOB, out-of-bag; ODI, oxygen desaturation index; OSA, obstructive sleep apnea; PCA, principal component analysis; PPI, pulse-to-pulse interval; PSG, polysomnography; PSQI, Pittsburgh Sleep Quality Index; RF, random forest; RNN, recurrent neural network; Sev, severe OSA; SpO₂, peripheral oxygen saturation; SVC, support vector classifier; SVM, support vector machine; Test, test set; Train, training set; Val, validation set; WC, waist circumference; XGBoost, extreme gradient boosting. Study-specific model and cohort labels are reported as used in the original studies.

**Table S3.** QUADAS-2 quality assessment of the included studies.

|  |  | **Risk of bias** | | | |  | **Applicability concerns** | | |
| --- | --- | --- | --- | --- | --- | --- | --- | --- | --- |
|  | Study | Patient selection | Index test | Reference standard | Flow and timing |  | Patient selection | Index test | Reference standard |
| 1 | Alvarez, D., et al. 2020 | L | H | L | U |  | L | L | L |
| 2 | Behar, J. A., et al. 2019 | L | H | L | U |  | L | H | L |
| 3 | Bozkurt, S., et al. 2017 | L | H | L | U |  | L | U | L |
| 4 | Cajal, D., et al. 2024 | U | H | L | L |  | H | H | L |
| 5 | Chen, J.-W., et al. 2023 | U | L | U | H |  | L | L | L |
| 6 | Chen, Q., et al. 2023 | L | H | L | L |  | L | L | L |
| 7 | Cho, S.-W., et al. 2022 | U | H | L | H |  | L | L | L |
| 8 | Dai, R., et al. 2024 | U | L | L | L |  | U | L | L |
| 9 | Ding, L., et al. 2024 | H | H | L | L |  | H | L | L |
| 10 | Ding, Y., et al. 2021 | L | L | L | H |  | H | L | L |
| 11 | dos Santos, R. R., et al. 2025 | U | L | L | L |  | L | U | L |
| 12 | Elwali, A., et al. 2019 | H | L | L | L |  | L | L | L |
| 13 | Fang, L., et al. 2025 | H | U | L | L |  | H | L | L |
| 14 | Ge, S., et al. 2024 | L | L | L | U |  | L | L | L |
| 15 | Han, H., et al. 2023 | H | L | L | U |  | L | L | L |
| 16 | Han, S. C., et al. 2024 | H | L | L | L |  | U | L | L |
| 17 | Hanif, U., et al. 2021 | L | L | L | U |  | L | L | L |
| 18 | He, S., et al. 2022 | L | U | L | H |  | U | L | L |
| 19 | Holfinger, S., J., et al. 2022 | L | H | L | L |  | L | H | L |
| 20 | Huang, W., et al. 2020 | L | L | L | L |  | L | L | L |
| 21 | Huo, J., et al. 2022 | U | U | L | L |  | L | L | L |
| 22 | Juang, C., et al. 2021 | H | H | L | L |  | H | L | L |
| 23 | Juang, C.-F., et al. 2023 | L | L | L | L |  | U | L | L |
| 24 | Kang, C., et al. 2023 | H | H | L | L |  | H | H | L |
| 25 | Keshavarz, Z., et al. 2020 | H | U | L | U |  | U | L | L |
| 26 | Kim, D., et al. 2026 | U | H | L | U |  | H | L | L |
| 27 | Kim, J., et al. 2017 | H | H | L | U |  | U | H | L |
| 28 | Kim, J., et al. 2024 | H | H | U | U |  | L | L | L |
| 29 | Kuan, Y.-C., et al. 2022 | L | H | U | U |  | L | H | U |
| 30 | Kuo, N.-Y., et al. 2024 | H | H | L | L |  | L | U | L |
| 31 | Leong, Z.H., et al. 2025 | U | L | L | L |  | L | L | L |
| 32 | Li, B., et al. 2025 | U | H | L | L |  | L | H | L |
| 33 | Li, C., et al. 2025 | U | L | L | U |  | L | L | L |
| 34 | Li, Z., et al. 2021 | H | H | L | L |  | U | L | L |
| 35 | Liu, T., et al. 2026 | L | H | L | U |  | L | U | L |
| 36 | Liu, W.-T., et al. 2017 | U | H | H | L |  | L | H | L |
| 37 | Molnár, V., et al. 2023 | U | L | L | H |  | L | L | L |
| 38 | Monna, F., et al. 2022 | H | U | L | U |  | U | U | L |
| 39 | Peng, D., et al. 2024 | L | L | L | L |  | L | L | L |
| 40 | Ramesh, J., et al. 2021 | U | L | L | L |  | L | L | L |
| 41 | Ramesh, J., et al. 2025 | U | H | L | U |  | L | H | L |
| 42 | Retamales, G., et al. 2024 | U | H | L | U |  | L | H | L |
| 43 | Rolón, R.E., et al. 2017 | U | H | H | U |  | H | H | L |
| 44 | Song, Y., et al. 2025 | U | L | L | L |  | H | L | L |
| 45 | Su, Z., et al. 2023 | U | L | L | U |  | L | L | L |
| 46 | Tsai, C., et al. 2022 | L | L | L | U |  | L | L | L |
| 47 | Ustun, B., et al. 2016 | L | H | L | L |  | U | U | L |
| 48 | Wang, B., et al. 2022 | L | H | L | L |  | L | L | L |
| 49 | Wang, K.-J., et al. 2016 | L | L | L | U |  | L | L | L |
| 50 | Weng, P., et al. 2022 | H | H | L | L |  | H | L | L |
| 51 | Xie, J., et al. 2023 | U | H | L | L |  | L | L | L |
| 52 | Xu, M., et al. 2026 | U | U | L | L |  | L | L | L |
| 53 | Yoo, H., et al. 2026 | U | H | L | L |  | L | L | L |
| 54 | Yook, S., et al. 2024 | U | L | L | L |  | L | L | L |
| 55 | Yue, H., et al. 2021 | U | L | L | U |  | U | L | L |
| 56 | Yue, H., et al. 2023 | U | L | L | L |  | L | L | L |
| 57 | Zhang, C., et al. 2024 | U | H | L | U |  | L | H | L |
| 58 | Zhang, Y., et al. 2025 | U | U | L | U |  | L | L | L |
| 59 | Zhu, Q., et al. 2026 | U | L | L | L |  | L | L | L |
| 60 | Zhu, X., et al. 2025 | U | H | L | U |  | U | H | L |

**Table S4.** Exploratory Subgroup Analysis of AI-Based OSA Screening Performance Using Non-PSG-Derived Inputs Across AHI Thresholds.

|  | N | AUC | Pooled Se (95% CI) | τ² logit(Se) | *P* value (Se) | Pooled Sp (95% CI) | τ² logit(FPR) | *P* value (Sp) |
| --- | --- | --- | --- | --- | --- | --- | --- | --- |
| **AHI ≥5 events/h (n= 14)** |  |  |  |  |  |  |  |  |
| **Region** |  |  |  |  |  |  |  |  |
| Asian | 10 | 0.917 | 0.92 (0.87–0.96) | 0.745 | .870 | 0.69 (0.47–0.84) | 1.723 | .887 |
| Non-Asian | 4 | 0.789 | 0.91 (0.68–0.98) | 2.311 |  | 0.67 (0.55–0.77) | 0.207 |  |
| **Data accessibility** |  |  |  |  |  |  |  |  |
| Public | 12 | 0.898 | 0.92 (0.86–0.96) | 1.123 | NA | 0.66 (0.50–0.79) | 1.196 | NA |
| Proprietary | 2 | NA | NA | NA |  | NA | NA |  |
| **Data source** |  |  |  |  |  |  |  |  |
| Community | 3 | NA | NA | NA | NA | NA | NA | NA |
| Hospital | 11 | 0.917 | 0.93 (0.86–0.97) | 1.434 |  | 0.70 (0.51–0.84) | 1.551 |  |
| **Modal type** |  |  |  |  |  |  |  |  |
| Unimodal | 12 | 0.904 | 0.91 (0.84–0.95) | 0.986 | NA | 0.71 (0.54–0.84) | 1.447 | NA |
| Multimodal | 2 | NA | NA | NA |  | NA | NA |  |
| **Algorithmic framework** |  |  |  |  |  |  |  |  |
| Deep learning | 5 | 0.957 | 0.95 (0.91–0.97) | 0.000 | .150 | 0.81 (0.73–0.88) | 0.000 | .056 |
| Machine learning | 9 | 0.871 | 0.90 (0.81–0.95) | 1.289 |  | 0.63 (0.42–0.80) | 1.553 |  |
| **Validation methods**^a^ |  |  |  |  |  |  |  |  |
| Internal validation | 10 | 0.889 | 0.93 (0.85–0.97) | 1.362 | NA | 0.62 (0.43–0.79) | 1.353 | NA |
| External validation | 3 | NA | NA | NA |  | NA | NA |  |
| **AHI ≥15 events/h (n= 22)** |  |  |  |  |  |  |  |  |
| **Region**^b^ |  |  |  |  |  |  |  |  |
| Asian | 15 | 0.884 | 0.87 (0.82–0.90) | 0.318 | .023 | 0.74 (0.64–0.82) | 0.732 | .709 |
| Non-Asian | 6 | 0.829 | 0.78 (0.70–0.84) | 0.159 |  | 0.77 (0.61–0.88) | 0.740 |  |
| **Data accessibility**^c^ |  |  |  |  |  |  |  |  |
| Public | 17 | 0.876 | 0.85 (0.81–0.88) | 0.214 | .316 | 0.75 (0.66–0.82) | 0.634 | .428 |
| Proprietary | 4 | 0.864 | 0.79 (0.63–0.89) | 0.546 |  | 0.80 (0.67–0.89) | 0.425 |  |
| **Data source** |  |  |  |  |  |  |  |  |
| Community | 8 | 0.836 | 0.83 (0.76–0.88) | 0.341 | .517 | 0.67 (0.54–0.79) | 0.637 | .151 |
| Hospital | 14 | 0.890 | 0.85 (0.80–0.89) | 0.303 |  | 0.78 (0.69–0.85) | 0.611 |  |
| **Modal type** |  |  |  |  |  |  |  |  |
| Unimodal | 18 | 0.872 | 0.84 (0.80–0.88) | 0.364 | .655 | 0.75 (0.65–0.82) | 0.849 | .846 |
| Multimodal | 4 | 0.847 | 0.86 (0.80–0.90) | 0.000 |  | 0.74 (0.68–0.79) | 0.000 |  |
| **Algorithmic framework** |  |  |  |  |  |  |  |  |
| Deep learning | 7 | 0.892 | 0.90 (0.88–0.91) | 0.002 | <.001 | 0.80 (0.61–0.91) | 1.275 | .396 |
| Machine learning | 15 | 0.848 | 0.82 (0.77–0.87) | 0.339 |  | 0.72 (0.64–0.79) | 0.491 |  |
| **Validation methods**^a^ |  |  |  |  |  |  |  |  |
| Internal validation | 13 | 0.864 | 0.86 (0.82–0.89) | 0.213 | .435 | 0.71 (0.64–0.77) | 0.233 | .601 |
| External validation | 8 | 0.865 | 0.83 (0.75–0.89) | 0.412 |  | 0.75 (0.57–0.87) | 1.240 |  |
| **AHI ≥30 events/h (n= 16)** |  |  |  |  |  |  |  |  |
| **Region** |  |  |  |  |  |  |  |  |
| Asian | 12 | 0.891 | 0.82 (0.75–0.87) | 0.392 | .340 | 0.84 (0.76–0.89) | 0.647 | .780 |
| Non-Asian | 4 | 0.947 | 0.92 (0.63–0.99) | 3.049 |  | 0.87 (0.61–0.96) | 1.612 |  |
| **Data accessibility** |  |  |  |  |  |  |  |  |
| Public | 13 | 0.889 | 0.80 (0.73–0.86) | 0.378 | NA | 0.86 (0.78–0.91) | 0.822 | NA |
| Proprietary | 3 | NA | NA | NA |  | NA | NA |  |
| **Data source** |  |  |  |  |  |  |  |  |
| Community | 4 | 0.905 | 0.83 (0.73–0.90) | 0.328 | .717 | 0.85 (0.74–0.91) | 0.421 | .911 |
| Hospital | 12 | 0.894 | 0.81 (0.72–0.88) | 0.591 |  | 0.85 (0.75–0.92) | 1.068 |  |
| **Modal type** |  |  |  |  |  |  |  |  |
| Unimodal | 15 | 0.896 | 0.83 (0.77–0.88) | 0.396 | NA | 0.83 (0.76–0.89) | 0.633 | NA |
| Multimodal | 1 | NA | NA | NA |  | NA | NA |  |
| **Algorithmic framework** |  |  |  |  |  |  |  |  |
| Deep learning | 6 | 0.913 | 0.90 (0.87–0.92) | 0.049 | <.001 | 0.84 (0.65–0.93) | 1.138 | .834 |
| Machine learning | 10 | 0.863 | 0.76 (0.69–0.82) | 0.268 |  | 0.85 (0.77–0.91) | 0.674 |  |
| **Validation methods** |  |  |  |  |  |  |  |  |
| Internal validation | 12 | 0.872 | 0.80 (0.72–0.86) | 0.421 | .265 | 0.82 (0.73–0.88) | 0.680 | .212 |
| External validation | 4 | 0.936 | 0.86 (0.75–0.93) | 0.430 |  | 0.90 (0.77–0.96) | 0.876 |  |

Abbreviations: FPR, false-positive rate; NA, not applicable; Se, sensitivity; Sp, specificity; τ², between-study variance.
ᵃSubgroup totals may differ from overall totals owing to missing validation methods data.

^b^Subgroup totals may differ from overall totals owing to missing region data.

^c^Subgroup totals may differ from overall totals owing to missing data accessibility data.

NA indicates that pooled estimates were not calculated because the bivariate random-effects model required at least 4 studies.

**Table S5.** Exploratory Subgroup Analysis of AI-Based OSA Screening Performance Using PSG-Derived Inputs Across AHI Thresholds.

|  | N | AUC | Pooled Se (95% CI) | τ² logit(Se) | P value (Se) | Pooled Sp (95% CI) | τ² logit(FPR) | P value (Sp) |
| --- | --- | --- | --- | --- | --- | --- | --- | --- |
| **AHI ≥5 events/h (n= 17)** |  |  |  |  |  |  |  |  |
| **Region**^b^ |  |  |  |  |  |  |  |  |
| Asian | 10 | 0.947 | 0.95 (0.95–0.96) | 0.003 | .452 | 0.81 (0.68–0.89) | 0.698 | .580 |
| Non-Asian | 5 | 0.920 | 0.93 (0.84–0.97) | 1.089 |  | 0.76 (0.60–0.87) | 0.613 |  |
| **Data accessibility** |  |  |  |  |  |  |  |  |
| Public | 12 | 0.962 | 0.95 (0.91–0.98) | 1.062 | .603 | 0.84 (0.72–0.91) | 1.043 | .341 |
| Proprietary | 5 | 0.961 | 0.96 (0.95–0.97) | 0.117 |  | 0.76 (0.60–0.87) | 0.535 |  |
| **Data source**^d^ |  |  |  |  |  |  |  |  |
| Community | 6 | 0.939 | 0.95 (0.93–0.97) | 0.372 | .898 | 0.70 (0.57–0.80) | 0.460 | .017 |
| Hospital | 9 | 0.923 | 0.95 (0.90–0.98) | 0.725 |  | 0.86 (0.77–0.92) | 0.043 |  |
| **Modal type** |  |  |  |  |  |  |  |  |
| Unimodal | 5 | 0.962 | 0.96 (0.94–0.97) | 0.174 | .951 | 0.80 (0.68–0.88) | 0.331 | .667 |
| Multimodal | 12 | 0.963 | 0.96 (0.92–0.98) | 1.063 |  | 0.83 (0.70–0.91) | 1.157 |  |
| **Algorithmic framework** |  |  |  |  |  |  |  |  |
| Deep learning | 10 | 0.974 | 0.97 (0.96–0.98) | 0.178 | <.001 | 0.86 (0.73–0.93) | 1.036 | .218 |
| Machine learning | 6 | 0.924 | 0.92 (0.86–0.95) | 0.430 |  | 0.76 (0.61–0.86) | 0.643 |  |
| **Validation methods** |  |  |  |  |  |  |  |  |
| Internal validation | 8 | 0.928 | 0.92 (0.87–0.95) | 0.374 | .002 | 0.79 (0.68–0.86) | 0.364 | .783 |
| External validation | 9 | 0.972 | 0.97 (0.96–0.98) | 0.354 |  | 0.81 (0.65–0.91) | 1.274 |  |
| **AHI ≥15 events/h (n= 15)** |  |  |  |  |  |  |  |  |
| **Region**^b^ |  |  |  |  |  |  |  |  |
| Asian | 9 | 0.934 | 0.88 (0.82–0.92) | 0.301 | .266 | 0.90 (0.80–0.95) | 1.013 | .378 |
| Non-Asian | 5 | 0.942 | 0.92 (0.86–0.95) | 0.343 |  | 0.85 (0.79–0.90) | 0.187 |  |
| **Data accessibility** |  |  |  |  |  |  |  |  |
| Public | 10 | 0.963 | 0.92 (0.84–0.96) | 1.185 | .446 | 0.91 (0.81–0.96) | 1.158 | .467 |
| Proprietary | 5 | 0.930 | 0.89 (0.84–0.93) | 0.240 |  | 0.86 (0.73–0.94) | 0.933 |  |
| **Data source**^d^ |  |  |  |  |  |  |  |  |
| Community | 6 | 0.939 | 0.86 (0.79–0.91) | 0.390 | .071 | 0.90 (0.83–0.94) | 0.418 | .368 |
| Hospital | 8 | 0.931 | 0.92 (0.89–0.94) | 0.048 |  | 0.85 (0.71–0.93) | 0.930 |  |
| **Modal type** |  |  |  |  |  |  |  |  |
| Unimodal | 4 | 0.951 | 0.90 (0.83–0.95) | 0.324 | .993 | 0.90 (0.79–0.96) | 0.670 | .673 |
| Multimodal | 11 | 0.947 | 0.90 (0.84–0.94) | 0.740 |  | 0.88 (0.78–0.93) | 1.007 |  |
| **Algorithmic framework** |  |  |  |  |  |  |  |  |
| Deep learning | 8 | 0.970 | 0.92 (0.87–0.96) | 0.481 | .206 | 0.94 (0.88–0.97) | 0.861 | .003 |
| Machine learning | 7 | 0.907 | 0.88 (0.81–0.93) | 0.417 |  | 0.79 (0.69–0.87) | 0.405 |  |
| **Validation methods** |  |  |  |  |  |  |  |  |
| Internal validation | 7 | 0.918 | 0.89 (0.85–0.93) | 0.204 | .475 | 0.79 (0.70–0.86) | 0.311 | .003 |
| External validation | 8 | 0.976 | 0.93 (0.83–0.97) | 1.618 |  | 0.95 (0.88–0.98) | 1.117 |  |
| **AHI ≥30 events/h (n= 13)** |  |  |  |  |  |  |  |  |
| **Region**^b^ |  |  |  |  |  |  |  |  |
| Asian | 9 | 0.843 | 0.81 (0.79–0.84) | 0.005 | NA | 0.96 (0.92–0.98) | 0.704 | NA |
| Non-Asian | 3 | NA | NA | NA |  | NA | NA |  |
| **Data accessibility** |  |  |  |  |  |  |  |  |
| Public | 9 | 0.956 | 0.86 (0.81–0.90) | 0.180 | .970 | 0.95 (0.92–0.97) | 0.340 | .559 |
| Proprietary | 4 | 0.967 | 0.86 (0.75–0.93) | 0.527 |  | 0.96 (0.92–0.98) | 0.655 |  |
| **Data source**^d^ |  |  |  |  |  |  |  |  |
| Community | 4 | 0.964 | 0.84 (0.74–0.91) | 0.353 | .962 | 0.96 (0.93–0.98) | 0.493 | .829 |
| Hospital | 7 | 0.927 | 0.84 (0.78–0.89) | 0.102 |  | 0.96 (0.91–0.98) | 0.751 |  |
| **Modal type** |  |  |  |  |  |  |  |  |
| Unimodal | 5 | 0.963 | 0.89 (0.84–0.93) | 0.221 | .126 | 0.96 (0.92–0.98) | 0.591 | .860 |
| Multimodal | 8 | 0.951 | 0.83 (0.77–0.88) | 0.242 |  | 0.96 (0.92–0.98) | 0.484 |  |
| **Algorithmic framework** |  |  |  |  |  |  |  |  |
| Deep learning | 8 | 0.971 | 0.86 (0.79–0.90) | 0.294 | .728 | 0.97 (0.95–0.98) | 0.313 | .257 |
| Machine learning | 5 | 0.933 | 0.84 (0.77–0.89) | 0.165 |  | 0.94 (0.88–0.98) | 0.658 |  |
| **Validation methods** |  |  |  |  |  |  |  |  |
| Internal validation | 6 | 0.927 | 0.84 (0.78–0.88) | 0.107 | .555 | 0.94 (0.89–0.97) | 0.525 | .228 |
| External validation | 7 | 0.971 | 0.86 (0.79–0.91) | 0.338 |  | 0.97 (0.95–0.98) | 0.358 |  |

Abbreviations: FPR, false-positive rate; NA, not applicable; Se, sensitivity; Sp, specificity; τ², between-study variance.
^b^Subgroup totals may differ from overall totals owing to missing region data.

^d^Subgroup totals may differ from overall totals owing to missing data source data.

NA indicates that pooled estimates were not calculated because the bivariate random-effects model required at least 4 studies.
